# Accurate Neural Network Fine-Tuning Approach for Transferable Ab Initio Energy Prediction across Varying Molecular and Crystalline Scales

**DOI:** 10.1021/acs.jctc.4c01261

**Published:** 2025-02-04

**Authors:** Wai-Pan Ng, Zili Zhang, Jun Yang

**Affiliations:** † Department of Chemistry, The University of Hong Kong, Hong Kong 999077, P. R. China; ‡ Hong Kong Quantum AI Lab Limited, Hong Kong 999077, P. R. China

## Abstract

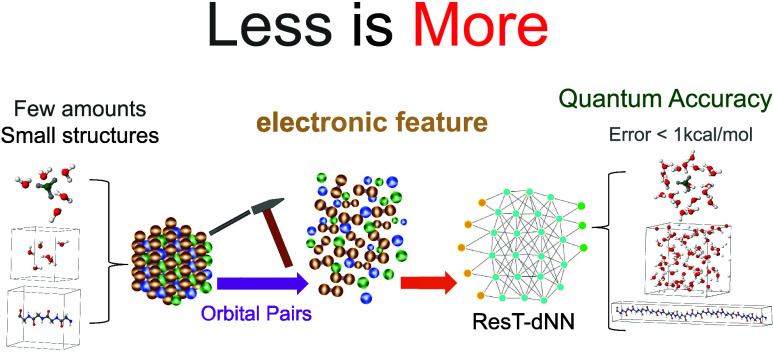

Existing machine learning models attempt to predict the energies of large molecules by training small molecules, but eventually fail to retain high accuracy as the errors increase with system size. Through an orbital pairwise decomposition of the correlation energy, a pretrained neural network model on hundred-scale data containing small molecules is demonstrated to be sufficiently transferable for accurately predicting large systems, including molecules and crystals. Our model introduces a residual connection to explicitly learn the pairwise energy corrections, and employs various low-rank retraining techniques to modestly adjust the learned network parameters. We demonstrate that with as few as only one larger molecule retraining the base model originally trained on only small molecules of (H_2_O)_6_, the MP2 correlation energy of the large liquid water (H_2_O)_64_ in a periodic supercell can be predicted at chemical accuracy. Similar performance is observed for large protonated clusters and periodic poly glycine chains. A demonstrative application is presented to predict the energy ordering of symmetrically inequivalent sublattices for distinct hydrogen orientations in the ice XV phase. Our work represents an important step forward in the quest for cost-effective, highly accurate and transferable neural network models in quantum chemistry, bridging the electronic structure patterns between small and large systems.

## 1 Introduction

Quantum chemistry has been an increasingly important tool for aiding molecular-level understanding of complex problems in chemistry, biological and materials sciences. A long-lasting challenge has been to accurately describe correlated electron interactions that are ubiquitously present in macromolecules.^1−5^ Popular computational models such as density functional theory (DFT) and other empirical calculations are useful yet often insufficient for treating such prominent problems. Although the modern advancement of *ab initio* quantum chemistry has enabled large-scale computations that were previously impossible at high-level quantum accuracy, the compositional, conformational and functional heterogeneity of molecular structure domains prevents these methods from carrying out high-throughput in silico navigation of novel molecules. Fortunately, the data-driven machine learning (ML) paradigms open a promising avenue to expedite molecular prediction without explicitly solving the Schrödinger equation, and have witnessed great success in a plethora of real-world applications.^6,7^

However, the size and performance of large-scale ML models are prevalently guided by an empirical scaling law on big data, including large languages models,^8−11^ ML-powered materials discovery^12,13^ and structural biology,^14^ which took decades to prepare versatile and valid data sets. It is strongly believed that there is a trade-off between ML data efficiency and its predictive capability, that is, the ML model with fewer data is less accurate than those trained on more data.^15^ However, in the era of AI for quantum chemistry, the acquisition and training of large-scale quantum chemistry data inevitably strain the computing power with extra computational costs which may become more expensive than solving electronic structure equations, and the ML superiority over direct calculations is lost when generating reference data sets containing many large molecules.

Moving beyond the DFT level of theory, the computational cost of highly accurate *ab initio* quantum chemistry methods increases rapidly with system size and thus it is desirable to have a better scaling of the model performance with data size. The recent investigations by us^16^ and others^17−23^ have shown that accurate ML models are feasible by training significantly reduced data points with judiciously supplied physical features, high-level *ab initio* reference data and novel learning infrastructures. For instance, the incorporation of cheap electronic structure information has been demonstrated to considerably improve ML models in predicting the energies and other molecular properties of new molecules unseen in training data sets.^7,16,23−26^ Our previous work^16^ demonstrated excellent energy accuracy of 0.30 kcal/mol for coupled-cluster prediction of all remaining molecules in the QM9 data set which contains 134 kilo drug-like molecules,^27^ by training a neural network model on almost one million electron pair energies decomposed from the energies of only 3000 randomly chosen molecules, which is significantly fewer than existing machine learning methods.

As compared to the electron-based feature design in a parsimonious and structured manner, what is the major limitation of the existing atom-based models, such as machine learning interatomic potentials (MLIPs)? MLIPs typically adopt an atomic energy decomposition ansätz in which the total energy of a molecule is divided into atomwise contributions. However, these individual contributions have not been explicitly supervised with *ab initio* reference values. Examples include the Behler-Parrinello neural networks,^28^ FCHL method,^29,30^ those based on Gaussian moments as physically inspired molecular descriptors (GMNN),^31^ as well as SOAP,^32^ SNAP,^33^ ANI^34^ and DeepMD.^35^ More recently, there are also message passing graph neural networks^36^ which require no handcrafted descriptors (e.g., SchNet,^37^ PhysNet,^38^ DimeNet,^39^ PAINN,^40^ NequIP^41^ and CHGNet^42^), and the descriptors from tight-binding calculations (OrbNet-Equi).^23^ NeuralXC which learns an accurate exchange-correlation (XC) functional,^43^ and DeePKS which learns a correction functional to a cheap DFT functional,^44,45^ follow the same energy decomposition. This atomic energy decomposition was invented to make ML models size-extensive and applicable to various molecular sizes for fast end-to-end prediction. Instead of making the atomic energy decomposition, the sGDML^46,47^ model works in the symmetrized gradient domain, in which the force can be unambiguously assigned to each atom. It has been found that, despite the total energy appearing to be trained accurately, the atomic energies suffer from an incorrect *ad-hoc* mapping.^48^ Only recently, a theoretical decomposition for atomic energies has been proposed within mean-field Hartree–Fock theory (HF) and DFT,^49−51^ in which the atomic energies are used as explicit learning targets but an error accumulation of the individual atomic energy predictions renders it uncompetitive with contemporary MLIPs. Moreover, the decomposed atomic energies most likely inherit systematic errors from DFT functionals.

Similarly to MLIPs, there are atom pairwise schemes with both early kernel based models^52,53^ and later neural network models such as BIM-NN,^54^ APNet,^55^ and the local equivariant graph neural network Allegro,^56^ which feature an atom pairwise decomposition of the total energy in the network. The idea of atom pairs has been also explored in ML to reconstruct the pairwise Hamiltonian elements either directly^57−60^ or indirectly.^61−63^ The resulting Hamiltonian is then diagonalized to reproduce the eigenvalues, atomic charges or other physical observables. Unlike in the case of effective single-particle Hamiltonian matrices whose elements can be mapped to atom pairs, there are no known mappings between atom pairs and “bond-energies”. In practice, these individual contributions are inferred from data^54^ during the training to match the total energy, and thus the direct approach suffers from error accumulation of individual elements. Even though our previous^16^ and current work rely on predicting pairwise correlation energies, they do not suffer from the accumulation of errors due to cancellation arising from a symmetrically distributed error distribution after explicitly training or fine-tuning target pairwise correlation energies.

In this work, we demonstrate that our previous transferable deep neural network (T-dNN) model^16^ with electronic descriptors and pair energy outputs can be pretrained on hundreds of small molecules to well generalize a representation for molecules and crystals of different sizes. We exploit various modern techniques for boosting a pretrained T-dNN model for quantum chemistry prediction of large systems at chemical accuracy (1 kcal/mol) or better, requiring only a single molecule for fine-tuning. We incorporate a residual connection, inspired by ResNet,^64^ to learn an energy correction of each electron pair, as well as low-rank fine-tuning techniques^65,66^ to adjust the pretrained weights. In certain cases, the fine-tuned model drastically improves the performance of the T-dNN base model by an order of magnitude, with no error accumulation from individual pair predictions. The subsequent architectural analysis reveals that it suffices to only marginally adjust the pairwise mapping encoded in the model parameters.

## 2 Methods

In this section, the electronic structure and NN methodologies are briefly presented. First, the generation of transferable electronic features for the T-dNN base model is reviewed. Second, the T-dNN network energy functional is introduced. Finally, the design of the residual transfer learning is discussed. The following notation has been adopted for labeling molecular orbitals: *i,j,k*,··· for localized molecular orbitals (LMOs), *a,b,c*,··· for canonical virtual orbitals, and *α,β,γ*,··· for atomic orbitals (AO).

### 2.1 T-dNN Feature Design

The key to designing transferable features of the T-dNN base model is to utilize a compact particle-hole excitation subspace that can automatically adapt to varying electronic environments in different molecules. To this end, we adopt an orbital-specific-virtual (OSV) representation^67^ which compresses and reparametrizes the global electron correlation pattern over the entire molecule into the local environment specific to each LMO, thanks to the orbital invariance in theory.^68^ For details regarding the OSV generation, see Section 2.2. In particular, the first-order interacting subspace Ψ^(1)^ is explicitly parametrized such that electrons in each LMO pair can also correlate in each other’s OSV domains, resulting in decomposed subspaces with four different excitation characters,
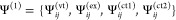
1from which individual amplitudes **T̃**_*ij*_^{vt,ex,ct1,ct2}^ = {*t̃*_*ij*_^μ̅_*i*_,ν̅_*j*_^, *t̃*_*ij*_^μ̅_*j*_,ν̅_*i*_^, *t̃*_*ij*_^μ̅_*i*_,ν̅_*i*_^, *t̃*_*ij*_^μ̅_*j*_,ν̅_*j*_^} are determined by eq 3. The set of feature amplitudes **T̃**_*ij*_^{vt,ex,ct1,ct2}^ collects the pseudoamplitudes which measure the importance of the electronic interactions of different characters, that is, *t̃*_*ij*_^μ̅_*i*_,ν̅_*j*_^ for OSV vertical excitation, *t̃*_*ij*_^μ̅_*j*_,ν̅_*i*_^ for OSV exchange excitation, as well as *t̃*_*ij*_^μ̅_*i*_,ν̅_*i*_^ and *t̃*_*ij*_^μ̅_*j*_,ν̅_*j*_^ for OSV charge transfer excitation in chemist’s notation. The OSV orbital set {| μ̅_*i*_⟩, | ν̅_*j*_⟩, | μ̅_*j*_⟩ and | ν̅_*i*_⟩} is introduced in the next section.

The input feature **x**_0_ of our T-dNN model is thus formulated as a set of pseudo energy tensors in a largely truncated OSV domain, i.e., its sum is a very crude approximation to the exact pair correlation energy yet sufficient in the data-driven ML context, which captures the feature of various interactions. Using a small number of compact OSV orbitals, the same as our previous work, this leads to a (4 × 8 × 8) feature tensor **x**_0_ = **ẽ**_**ij**_ for each LMO pair *ij*
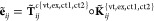
2where ◦ denotes the element-wise Hadamard product, and **T̃**_*ij*_^*c*^ = 2**T̃**_*ij*_ – **T̃**_*ij*_^†^ is the contravariant amplitude matrix in the compact OSV basis. The set of exchange integrals **K̃**_*ij*_^{vt,ex,ct1,ct2}^ = {*K̃*_*ij*_^μ̅_*i*_, ν̅_*j*_^, *K̃*_*ij*_^μ̅_*j*_, ν̅_*i*_^, *K̃*_*ij*_^μ̅_*i*_, ν̅_*i*_^, *K̃*_*ij*_^μ̅_*j*_, ν̅_*j*_^} contains the electronic interactions of different characters corresponding to the amplitudes *t̃*_*ij*_^μ̅_*i*_, ν̅_*j*_^, *t̃*_*ij*_^μ̅_*j*_, ν̅_*i*_^, *t̃*_*ij*_^μ̅_*i*_, ν̅_*i*_^ and *t̃*_*ij*_^μ̅_*j*_, ν̅_*j*_^. **T̃**_*ij*_^{vt,ex,ct1,ct2}^ is approximated in a way analogous to canonical MP2 amplitudes but in drastically reduced dimension
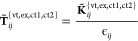
3where ϵ_*ij*_ = *f*_*ii*_ + *f*_*jj*_ – *f*_μ̅_*i*_ μ̅_*i*__ – *f*_ν̅_*j*_ ν̅_*j*__, and *f*_*ii*_ is the diagonal element of the Fock matrix in the LMO space, and similarly for *f*_μ̅_*i*_ μ̅_*i*__ in the OSV space. The resulting pseudo energy tensor **ẽ**_*ij*_ therefore respects physically distinct electron correlation characters which facilitate the mapping from LMOs to exact pair correlation energies. We refer readers to eqs 6–9 in Section 2 of our previous work for more details.^16^

The electronic features for periodic systems employs Bloch AO orbitals ϕ_α_^***k***^(***r***)
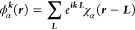
4where ***L*** is a translation vector of the unit cell and ***k*** a crystal momentum vector. In Γ-only mesh (***k*** = 0), the phase term *e*^*i****k***·***L***^ vanishes. The Γ-only pseudo energy feature tensor **ẽ**_*ij*_^Γ^ is thus formally similar to eq 2, with the pseudoamplitudes **T̃**_*ij*_^Γ{vt,ex,ct1,ct2}^ and the exchange integrals **K̃**_*ij*_^Γ{vt,ex,ct1,ct2}^ adapted with Γ-only Bloch AO integrals to account for periodic boundary condition.

### 2.2 Generation of OSV Subspaces

The OSV subspaces are constructed by performing a singular value decomposition (SVD) of the local MP2 semicanonical amplitudes *t*_*ii*_^*ab*^ specific for each LMO *i*
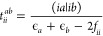
5where ϵ_*a*_ is the orbital energy of a canonical virtual orbital *a*, and (*ia*|*ib*) is the electron repulsion integral. The singular vector *Q*_*ia*_^μ̅_*i*_^ from this SVD serves as the orbital transformation matrix to rotate the virtual orbital space |*a*⟩ into a compact OSV representation | μ̅_*i*_⟩ which is specific to LMO *i*
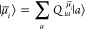
6which can be truncated based on the singular values, i.e., the singular vector *Q*_*ia*_^μ̅_*i*_^ with small singular values contributes little to the final amplitudes and can be neglected. The resulting OSV space is therefore only a selected subspace with the important *Q*_*ia*_^μ̅_*i*_^ from the canonical virtual space. Based on our previous experience,^16^ only a few OSVs with the largest singular values are sufficient to build the very sparse electronic descriptors to refactor the learning pattern in the OSV basis in which the critical correlation environment is compressed in the vicinity of the LMO.

### 2.3 Neural Network Energy Functional

The ubiquitous atomic decomposition of the total energy *E*_tot_ in atomistic machine learning reads
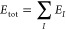
7where *E*_*I*_ denotes the atomic energy of atom *I* in a molecule. The atomic energies are further decomposed into atom pairwise contributions in recent works such as APNet^55^ and Allegro^56^
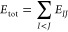
8The decomposition ansätzes implicitly encode some regularities and patterns in the architecture. However, *ad-hoc* mappings^48^ still exist as the loss function has only reference total energies instead of individual contributions. In *ab initio* wave function theory, the post-HF computation is usually performed to obtain the correlation energy (*E*_c_) of a molecule, following the HF energy (*E*_HF_) computation. The total molecular energy is given by
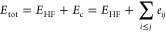
9*E*_c_ is further formulated according to the summation of the pairwise correlation energies *e*_*ij*_ associated with the occupied orbital pairs *ij*.

Previous electron-based ML models^16,18−22,24^ learn the LMO correlation energy pairs *e*_*ij*_, thanks to the availability of *ab initio* reference values of the latter. Our previous work T-dNN model^16^ acts as a universal neural network energy functional that maps the pseudo energy descriptors **ẽ**_*ij*_ to exact electron correlations *e*_*ij*_. As such, the correlation energy is predicted according to
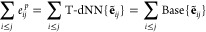
10where T-dNN is a fully connected feed-forward neural network or multilayer perceptron (MLP), which acts as a base model in the current work. In particular, T-dNN minimizes the following loss function, assuming Einstein sum
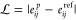
11without the need to explicitly supervise the total correlation energy predictions, since individual pairwise contributions are accurately predicted. In particular, separate models are used for training diagonal and off-diagonal pairs. For the latter, pairs with negligible contributions are screened and do not enter the training and prediction stage, see Section 3.1 for computational details.

**Figure 1:**
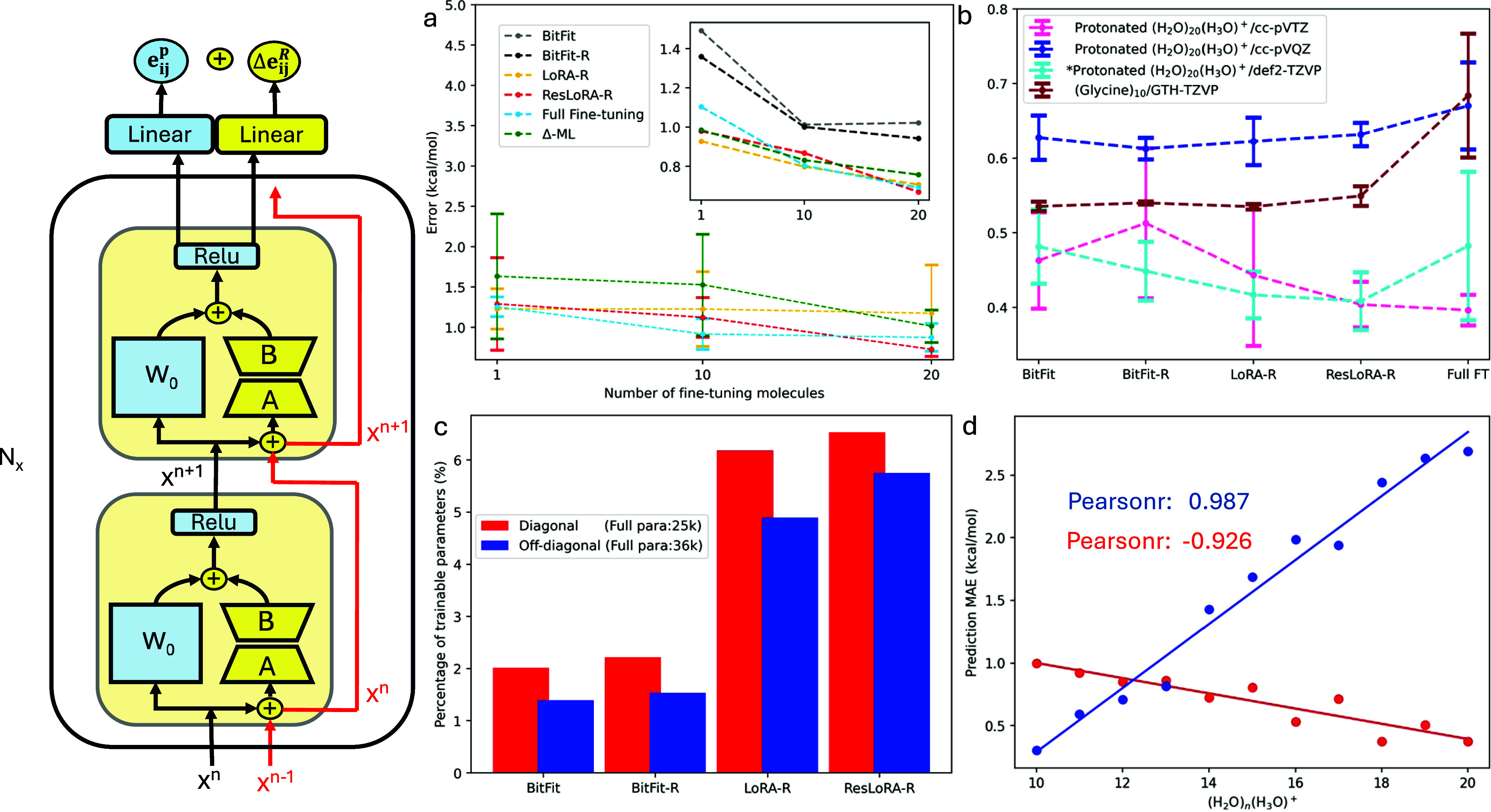
Left: Schematic illustration of various ResT-dNN architectures. The blue squared box indicates the T-dNN base model architecture. The LoRA blocks are indicated by A and B (yellow). Residual connections are added between the LoRA blocks (red). Together with the residual connection that explicitly learns Δ**e**_**ij**_^**R**^, the two resulting architectures are denoted as LoRA-R and ResLoRA-R respectively. (a) The mean and standard deviation of MAEs from six random repetitions for various fine-tuning methods on (H_2_O)_64_ with T-dNN pretrained on (H_2_O)_6_, and the inset shows the best model accuracy. (b) Performance of various fine-tuning methods on the protonated clusters and 1-D glycine chains with T-dNN pretrained on their smaller counterparts, with asterisk denoting CCSD(T) training level. (c) Trainable parameters of various ResT-dNN methods. (d) Prediction MAEs on protonated clusters of varying size with cc-pVTZ basis set for ResLoRA-R (red) fine-tuned on a single (H_2_O)_20_(H_3_O)^+^ cluster and the base T-dNN (blue).

T-dNN^16^ demonstrates that a single MLP suffices to learn an accurate and transferable mapping with closely related approaches based on XGBoostRegressor^18^ or Gaussian process regression,^19−22,24^ but with similar or less molecular training data albeit trained with a neural network. Besides, unlike other works, no manual or handcrafted feature selection or extraction among elements in **ẽ**_*ij*_ is carried out due to the correlation importance ranking of OSVs (eq 5) with important singular values; the regularization in network weight is unnecessary when the low number of feature descriptors (Section 2.1) is used in this study. As demonstrated to the base T-dNN model in our early work (Figure 1 of our ref (16)), the automatic selection of feature amplitudes by using not less than 4 OSVs shows that the mean absolute errors (MAEs) of predicted correlation energies for *n*-hexane and isobutane are consistently below 0.5 kcal/mol, as compared to the reference CCSD/cc-pVTZ results.

In addition, T-dNN can predict water clusters as large as (H_2_O)_64_ and (H_2_O)_128_ in absolute accuracy by training on nonperiodic small water clusters such as (H_2_O)_8_ and (H_2_O)_16_. It is observed that although there is retained accuracy, it can be anticipated for even larger systems that the errors will ultimately accumulate and considerably deteriorate the prediction accuracy if relying on solely small system training, if the errors are not expressed in a size-intensive metric, e.g., per atom, per H_2_O etc. We are uncomfortable with the metric trick, and thus in Section 2.4 we describe approaches to fine-tune the underlying pair energy functional with only a few molecular data for correcting the biased predictions. It is observed in Section 3 that the bias can be corrected and individual errors are canceled rather than accumulated. The use of MLP and an elegant set of pseudo energy tensors **ẽ**_*ij*_ might better benefit further exploration in realizing similar architectural building blocks for existing graph neural network interatomic potentials, in contrast to closely related works.

### 2.4 Residual Transfer Learning

Our T-dNN model is set up with the pseudo energy features relying on sufficiently compact LMOs in their local electronic environments via particle-hole excitations. For instance, as shown in Figure S1 in Supporting Information, the LMOs and OSVs are transferable and largely similar among different ice-XV morphologies of distinct sublattice structures.

When examining the factors that may limit the transferability of the T-dNN base model, it is important to realize that the transferable prediction of large molecules from small molecules is still vulnerable to the long-range electronic interaction arising from many remote electronic configurations that are apparently weaker and more system dependent than close configurations,^69^ which is not well represented in the training data consisting of small molecules. Nevertheless, as shown in Figure S6, the individual LMO-pair energy prediction errors for large systems such as (H_2_O)_64_ are actually quite small. Unlike MLIPs simply neglecting long-range interatomic interactions, the T-dNN predicts correlation energies of all kept LMO-pairs including those important long-range orbital pairs through pair preselection according to OSV orbital overlaps (eq 10 of our ref (16)) by transforming all canonical virtual orbitals. Thus, each orbital pair automatically spans a cluster of important atoms that are collectively mapped to the pair correlation energy by using the pseudo energy feature tensor (eq 2). This approach implies an importance of and equivalence to utilizing atomic domain-wise selection, as opposed to the popular atomwise selection, for MLIP-based descriptors. Therefore, we anticipate that the underlying universal mapping will manifest in delicate fine-tuning of the base T-dNN model when long-range interactions are taken into account, i.e., the magnitude and distribution of network weights and bias terms that encode the pairwise mapping would vary little despite that the total energies can be very different.

Bearing this in mind, we incorporated the residual connection^64^ and the low-rank fine-tuning^65,66^ techniques on top of the T-dNN model to boost the prediction transferability, rather than simply increasing data or model complexity. The performance of the residual transfer learning models, dubbed as ResT-dNN for a range of specific fine-tuning approaches as described below, will be demonstrated to both molecules and periodic crystalline solids. The base model, T-dNN, can be in principle replaced by various atomistic models containing atomwise MLP layers in the property prediction block such as SchNet^37^ and SchNOrb,^57^ as well as those containing atomistic pairwise MLP layers such as Allegro,^56^ among many others. Before delving deeper, the full fine-tuning in this work refers to training the base model without any modification in network architecture, but by adjusting all weights and bias.

First of all, a residual connection (R) is added from the last hidden layer to the output layer of the base model for learning pair energy corrections Δ**e**_**ij**_^**R**^, with learnable weight **W**^**R**^ and bias **b**^**R**^, as denoted in the yellow circle of the ResT-dNN architecture in Figure 1. Therefore, the fine-tuned pair correlation energy reads
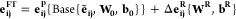
12where **e**_**ij**_^**P**^ is the predicted pair energy by the base model, and **W**_0_ and **b**_0_ are the pretrained weight and bias vectors of the base model, respectively. The training is supervised with the exact pair energy as seen in eq 16.

Second, we investigated the performance of low-rank adaptation (LoRA)^65^ for fine-tuning the T-dNN base model. For a given input vector **x**, which on the first layer is in the present study the pseudo energy feature tensors of fine-tuning molecules, the output is modified from T-dNN according to eq 13
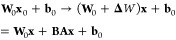
13For each pretrained layer with the frozen weight matrix **W**_0_, the weight adjustment Δ**W** is parametrized by the product of two learnable low-rank matrices **A** and **B**, which drastically reduces the number of parameters during fine-tuning. The resulting rank of **A** and **B** also naturally measures how much the pretrained representation needs to be adjusted when transferred to larger systems. Together with the residual connection that learns Δ**e**_**ij**_^**R**^, this method is denoted as LoRA-R.

Third, extra residual connections (Res) can be added to LoRA-R blocks featuring the input-shortcut structure to ensure better feature transmission along the hidden layers as well as gradient backpropagation in a deep neural network,^70^ which leads to ResLoRA-R. More specifically, the LoRA-R block at a given layer receives a modified input which is the sum of the inputs **x**^**n**–1^ and **x**^**n**^ of the previous (*n* – 1) and current (*n*) layers as follows
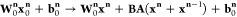
14with *n* indexing a given layer.

Finally, we have attempted to fine-tune the T-dNN base models by varying only the bias terms **b**_0_ using BitFit,^66^ with and without residual connection that learns Δ**e**_**ij**_^**R**^, which are termed as BitFit and BitFit-R, respectively
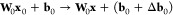
15This serves as a diagnosis for the ease of fine-tuning and whether it is necessary to fine-tune the weights which contain more parameters. For all cases, a loss function, i.e., the mean absolute error on pair predictions, which measures the deviation of the pair energy sum **e**_**ij**_^**P**^ + Δ**e**_**ij**_^**R**^ from the exact reference pair energy as labeled by **e**_**ij**_^**exact**^, is minimized
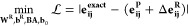
16where **W**^**R**^, **b**^**R**^, **A**, **B** and **b**_0_ are retrained depending on the specific fine-tuning methods to be used. In particular, the LoRA blocks and the residual connection that learns Δ**e**_**ij**_^**R**^ are retrained simultaneously. All these fine-tuning approaches (LoRA-R, ResLoRA-R, BitFit, BitFit-R), which are applied to the base T-dNN model, constitute the ResT-dNN family.

eq 16 suggests that ResT-dNN is different from traditional Δ-ML approaches that directly supervise and learn the difference between two different levels of theory, which in the context of the current work, corresponds to learning the differences between the exact pair energies and base model predictions. In Section 3.2, we also compare such Δ-ML approach which learns the mapping from **ẽ**_**ij**_ to (**e**_**ij**_^**exact**^ - **e**_**ij**_^**P**^).

## 3 Results

### 3.1 Computational Details

#### 3.1.1 Reference Electronic and Geometric Structures

The geometries of (H_2_O)_6_ clusters and (H_2_O)_64_ in periodic water boxes were obtained from the previous work^71^ by Cooper et al., resulting in around 800 (H_2_O)_6_ and 500 (H_2_O)_64_ randomly chosen to be used in this work. Meanwhile, the protonated clusters (H_2_O)_*n*_(H_3_O)^+^ were obtained from the Cambridge Cluster Database.^72,73^ For cluster sizes *n* = 10, 11, 12, 13, 14, 15, 16, 17, 18, 19, and 20, there are 74, 79, 113, 119, 108, 140, 121, 138, 114, 125, and 143 structures, respectively. The ice XV 2 × 2 × 2 supercell structures were obtained from a previous computational work on ice XV hydrogen orderings.^74^ There are 18 possible symmetrically distinct hydrogen orderings in the ice XV phase. We randomly chose one hydrogen ordering, i.e., ice XV-4B1, and sampled 1000 of its primitive cell structures from *ab initio* molecular dynamics (AIMD) trajectories at the PBE+D3 level of theory with GTH-SZV basis set, using a Nosé–Hoover thermostat with the NVT ensemble and at the temperature of 350 K and time step of 1 fs. The MD simulation was carried out with CP2K code,^75^ with a plane-wave cutoff of 300 eV and Γ point-only k-point mesh. Similarly, 800 (glycine)_4_ and 100 (glycine)_10_ were sampled, from the trajectory at the temperature of 300 K with other settings being the same as the ice.

The periodic and molecular HF calculations were carried out using PySCF,^76^ and the orbitals were then localized by the Pipek-Mezey localization scheme^77^ The feature preparation for **ẽ**_*ij*_ is then done according to Section 2.1 and 2.2 and the subsequent noncanonical molecular MP2 reference pair energies calculations were performed with our in-house implementation of OSV-MP2,^78,79^ and its adaption to the periodic implementation for periodic systems at the Γ point. A tight OSV cutoff threshold of 3.2 × 10^–5^ was applied, which screens out unimportant remote off-diagonal pairs with negligible loss in accuracy.^16,78^ In particular, an asymptotic cost analysis of feature computation can be found in Section 2.4 and Table 1 in our previous work.^16^ The baseline Hartree–Fock computation, which scales as *O*(*N*^4^), accounts for most of the computational time. Empirically, the computational time for fine-tuning decreases by lowering the LoRA rank (fewer tunable parameters), and we refer interested readers to the LoRA work^65^ for more details. It shall be noted that the model itself is not limited to learning MP2 energies, but also gold-standard coupled-cluster energies obtained with DLPNO–CCSD(T), implemented in ORCA program 5.0.3.^80^ The tight PNO cutoff was used for benchmark accuracy.

**Table 1:** A Table Summarizing the Systems, Levels of Theory and Basis Sets^a^

pretraining data	structures	800 (H_2_O)_6_		600 (Gly)_4_	800 ice XV-4B1 unit cells
	level of theory	MP2/GTH-DZVP			
fine-tuning data	structures	*n* (H_2_O)_64_ (*n* = 1, 10, 20)	129 (H_2_O)_*n*_(H_3_O)^+^ (*n* = 5, 6, 7, 8)	50 (Gly)_4_	1 ice XV-4B1 supercell
	level of theory	MP2/GTH-DZVP	MP2/cc-pVTZ	MP2/GTH-TZVP	MP2/GTH-DZVP
			MP2/cc-pVQZ		
			CCSD(T)/def2-TZVP		
	structures		1 (H_2_O)_20_(H_3_O)^+^	1 (Gly)_10_	
	level of theory		MP2/cc-pVTZ	MP2/GTH-TZVP	
			MP2/cc-pVQZ		
			CCSD(T)/def2-TZVP		
test data	structures	500 (H_2_O)_64_	143 (H_2_O)_20_(H_3_O)^+^	100 (Gly)_10_	18 distinct ice-XV supercells
	level of theory	MP2/GTH-DZVP	MP2/cc-pVTZ	MP2/GTH-TZVP	MP2/GTH-DZVP
			MP2/cc-pVQZ		
			CCSD(T)/def2-TZVP		

a The required pretraining and fine-tuning molecules and the sizes of the final test sets are also shown.

#### 3.1.2 Data Preparation for T-dNN

A simple base model was pretrained on 800 randomly chosen (H_2_O)_6_ clusters embedded in periodic boxes at periodic MP2 level of theory with the GTH-DZVP pseudopotential basis set. This base model was fine-tuned directly with 1, 10, and 20 liquid (H_2_O)_64_ conformations using the same level of theory and basis set. For predicting the energies of 143 large protonated clusters (H_2_O)_20_(H_3_O)^+^, the same pretrained T-dNN model on (H_2_O)_6_ was also first fine-tuned using 129 small protonated clusters (H_2_O)_5–8_(H_3_O)^+^ using molecular MP2 with both cc-pVTZ and cc-pVQZ basis sets, as well as CCSD(T) with def2-TZVP basis set, and subsequently fine-tuned using a single (H_2_O)_20_(H_3_O)^+^. For predicting 100 (Gly)_10_ using the GTH-TZVP basis set, another base model was trained with 600 1D chains of (Gly)_4_ with smaller GTH-DZVP, which was then first fine-tuned with 50 (Gly)_4_ with periodic MP2 level of theory and GTH-TZVP pseudopotential basis set, and further fine-tuned with a single (Gly)_10_. For predicting the energy of various ice XV 2 × 2 × 2 supercell structures to distinguish 18 hydrogen-ordered sublattices using periodic MP2 with GTH-DZVP basis set, our last base model was trained on the primitive cell of ice XV-4B1, sampling 800 different water conformations. The data preparation is also summarized in Table 1 in a more structured manner for better reference.

#### 3.1.3 Neural Network Training Details

In this work, feed-forward neural network models with 10 hidden layers (each with 50 neurons) were trained and used as the base models for various systems. In particular, diagonal and off-diagonal orbital pairs were trained separately. A batch size of 64 is applied for splitting orbital pairs. Prior to training, both input descriptors and output pair energies are standardized with scikit-learn.^81^ The loss function is computed as the mean absolute error of the pair energy deviations with equal weight applied to each pair. The ReLU activation function^82^ and the Adam optimizer^83^ for backpropagation were employed throughout the training for all architectures. Typically, the base models were trained for 1000 epochs with learning rates of 1 × 10^–3^ and 1 × 10^–4^. For the fine-tuning stage, the retraining was performed with 200 and 800 epochs at much smaller learning rates, i.e., 1 ×10^–5^ and 1 × 10^–6^, respectively. For the ice-XV systems, the fine-tuning was carried out with even smaller learning rates 1 × 10^–7^ and 5 × 10^–8^ for 800 epochs. The best results upon hyperparameter scan are presented in Supporting Information.

The **A** and **B** matrices in LoRA were initialized with zeros and heuniform,^84^ respectively. The zero initialization prevents an explosive training error at the beginning of the fine-tuning. This was similarly performed for the residual connection that explicitly learns Δ**e**_**ij**_^**R**^. All the architectural implementations were completed with Keras,^85^ with the sequential model responsible for building the base model and the functional API for various fine-tuning techniques.

### 3.2 MP2 Energy for Periodic Systems

The ability to leverage electron correlation information stored in smaller systems for transferable use in larger ones is truly fascinating, particularly given the challenges posed by the curse of dimensionality in traditional quantum chemistry methods and the rapid expansion of chemical space. In the context of periodic systems, we demonstrate the performance of our ResT-dNN models, which refine the T-dNN base model - pretrained on small water clusters (H_2_O)_6_ and short peptide chain (Gly)_4_ in periodic lattices. Remarkably, this model can accurately predict MP2 energies of both noncovalent and covalent periodic systems, including liquid water, represented by (H_2_O)_64_ configurations in unit cell, as well as for the chain conformations of longer (Gly)_10_.

The T-dNN base model’s prediction on (H_2_O)_64_ yields a rather large MAE of 33.8 kcal/mol. Conversely, when a single cluster conformation of (H_2_O)_64_ is trained directly with randomly initialized parameters, the MAE decreases to 13.4 kcal/mol. However, as illustrated in Figure 1a and Table S1 in Supporting Information, fine-tuning the base T-dNN model on a single cluster (H_2_O)_64_ remarkably reduces the prediction MAE on (H_2_O)_64_ by an order of magnitude. This significant improvement indicates that a substantial amount of useful knowledge is transferred through the learned weights in the pretrained T-dNN on 800 small (H_2_O)_6_ conformations.

The fine-tuning learning curves obtained from six random repetitions are shown in Figure 1a, clearly revealing that the performance and costs of models are highly correlated with the fine-tuning techniques and the number of fine-tuning molecules. Overall, the errors decrease monotonically as more target molecules of (H_2_O)_64_ are fine-tuned. When using BitFit (gray) to fine-tune the T-dNN bias terms for learning total pair correlation energies, the predictions do not achieve chemical accuracy (within 1 kcal/mol). However, incorporating the residual connection with BitFit-R (black) for explicitly learning the energy correction Δ**e**_**ij**_^**R**^ reduces the errors, although BitFit-R still requires retraining 20 (H_2_O)_64_ clusters to approach chemical accuracy. This implies that for this system, it is necessary to fine-tune the weights for better accuracy. For example, the LoRA-R (yellow) and ResLoRA-R (red) techniques yield MAEs below 1 kcal/mol with just one fine-tuning (H_2_O)_64_ cluster for the best model. By fine-tuning 20 (H_2_O)_64_ clusters, LoRA-R and ResLoRA-R boost the accuracy to 0.71 and 0.67 kcal/mol, respectively. Among all fine-tuning techniques, ResLoRA-R tends to reduce the variance of MAEs on the 500 (H_2_O)_64_ conformations with an increasing number of fine-tuning molecules. It shall be noted that moderately increasing OSVs (e.g., 4–8 OSVs in Table S3) not only improves the fine-tuned predictions, but also allows the models to benefit from more fine-tuning molecules for better accuracy.

Finally, we also investigate the Δ-ML approach (Figure 1a, green, and Table S2) for liquid water, it is found that, consistent with a recent work^86^ in general, the Δ-ML approach results in less stable models compared with transfer learning. Surprisingly, with the same pseudo energy tensors as descriptors, its predictions are still accurate albeit inferior to other low-rank fine-tuning approaches in terms of model variances and best model accuracy. We attribute this to the lack of knowledge transfer and a less rigorous descriptor to target mapping.

For the 1-D periodic glycine chain, where glycine units are covalently bonded, the T-dNN base model was pretrained on (Gly)_4_ using the GTH-DZVP basis set. It was then fine-tuned with a larger basis set, GTH-TZVP, using only 50 fine-tuning molecules of (Gly)_4_. As seen in Figure 1b and Table S4 in Supporting Information, without fine-tuning on any larger target system, the MAE for predicting (Gly)_10_/GTH-TZVP is already only 0.91 kcal/mol. Furthermore, fine-tuning with a single (Gly)_10_ molecule reduces the error to 0.53 kcal/mol, nearly halving it. Interestingly, tuning with BitFit and BitFit-R suffices for this system, potentially due to its relative ease, i.e., 1-D periodic, compared with other systems. Overall, for periodic systems, all low-rank approximations prove to be robust, with ResLoRA-R demonstrating lower error variance.

### 3.3 MP2 and CCSD(T) Molecular Energies

We examine the prediction accuracy of MP2 and gold-standard CCSD(T) energies for protonated water clusters, specifically (H_2_O)_20_(H_3_O)^+^, using various large basis sets. The positively charged (H_2_O)_20_(H_3_O)^+^ cluster presents a particular challenge for the T-dNN base model, when using the pretrained base model solely on neutral (H_2_O)_6_ clusters in unit cell with a small basis set GTH-DZVP. The prediction with the T-dNN base model yields the MAEs of hundreds of kcal/mol (Tables S5–S7 in Supporting Information). Our results demonstrate that minimal retraining efforts are sufficient to drastically improve the accuracy better than 1 kcal/mol.

Since the T-dNN base model has little knowledge about protonated clusters, we first fine-tune it with the full parameters on a mixture of 129 small protonated clusters, ranging from (H_2_O)_5_(H_3_O)^+^ to (H_2_O)_8_(H_3_O)^+^. Such full-parameter fine-tuning is not necessary when the base model is pretrained on more diverse but small systems in future. The resulting best model can already predict the MP2 energies of (H_2_O)_20_(H_3_O)^+^ with an MAE of only 0.52 kcal/mol at cc-pVQZ basis set. However, considering the large energy variance, we applied various ResT-dNN models to retrain only one (H_2_O)_20_(H_3_O)^+^ cluster (Figure 1b and Tables S5–S7 in Supporting Information).

In general, the ResT-dNN notably improves both accuracy and model variance, when compared with full-parameter fine-tuning. For cc-pVTZ, ResLoRA-R yields an MAE of 0.37 kcal/mol for the best fine-tuned model. We thus go on to demonstrate the MAEs of the ResLoRA-R prediction for other protonated clusters (H_2_O)_*n*_(H_3_O)^+^ (*n* = 10–20) by fine-tuning with a single (H_2_O)_20_(H_3_O)^+^ cluster (red line in Figure 1d). The prediction MAE tends to decrease linearly, within a drift of 0.2 kcal/mol, with respect to *n* that becomes increasingly similar with the fine-tuning (H_2_O)_20_(H_3_O)^+^. This deviation is an indicator for determining whether the model has to be retrained for the target system in a controllable accuracy threshold when embarking on different sizes. A linear yet opposite relationship is observed with only fine-tuning on the small protonated cluster (*n* = 5–8) (blue line in Figure 1d). Notably, by comparing the red and blue lines in Figure 1d with fine-tuning on the larger system, the generalization to various sizes improves, which is indicated by a smaller rate of error increase.

The error growth with system size justifies the necessity of fine-tuning large systems. Such a quasi-linear growth shown in Figure 1d results from loss of important correlations among longer-range orbital pairs that are not present in pretraining molecules, and the number of such long-range pairs typically increases linearly with cluster size.^78^ On the other hand, when the model is fine-tuned on clusters of size exceeding the orbital correlation length, we anticipate accurate prediction on much larger clusters than it was fined-tuned on.

Furthermore, we highlight that the gold-standard CCSD(T)/def2-TZVP energies can be accurately reproduced, with all resulting MAEs below 0.50 kcal/mol (Figure 1b, cyan and Table S7 in Supporting Information), using the pretrained T-dNN model on MP2/GTH-DZVP energy data. Here, various models are retrained with a small pool of CCSD(T)/def-TZVP energy data for small protonated water clusters, followed by fine-tuning a single (H_2_O)_20_(H_3_O)^+^. In particular, the low-rank ResLoRA-R model refines the prediction at an accuracy of 0.41 ± 0.04 kcal/mol, with the best model attaining 0.37 kcal/mol (Table S7 in Supporting Information). Our findings indicate that the effects of the basis set and theory level can be accurately captured in a single retraining step on small molecules and then transferring this knowledge to the prediction of large target systems requires substantially fewer data.

In all instances above, all ResT-dNN methods can achieve at least the same accuracy as the full-parameter fine-tuning model by adjusting only a small portion (1.5 to 6.5%) of the full trainable parameters (Figure 1c). Therefore, our models offer a highly cost-effective transfer learning approach, requiring only a modest amount of small training molecules, followed by very few larger molecules to achieve high accuracy. Notably, low-rank fine-tuning techniques with a single or a few fine-tuning molecules become especially valuable and preferred over full fine-tuning when *ab initio* methods such as CCSD(T), are intended for large systems, rather than DFT. This is in contrast to a recent work^87^ that studies dicalcium silicates with transfer learning, where full fine-tuning has been found essential with 8000 training DFT energies. Finally, we also find that fine-tuning can accurately reproduce the binding curves of charged–charged dimers from the BFdB database,^88−90^ see Figure S7 for details.

### 3.4 Ice-XV Sublattice Hydrogen Ordering

Here, we present a demonstrative application to the hydrogen ordering of ice XV sublattices. As new ice phases continue to be discovered, at least 20 crystalline ice phases had been identified by 2021.^91^ Determining the crystal structures is both experimentally and theoretically challenging, due to the presence of competing ice polymorphs, which necessitates the development of highly accurate *ab initio* methods to distinguish them.^92^ In this work, we studied the ice XV phase, a hydrogen-ordered polymorph of ice VI, which has been the subject of experimental discrepancies and theoretical disputes,^74,93^ as Ice XV features 18 possible structures with different hydrogen orderings. The hydrogen bonding networks can be arranged in three distinct configurations, denoted as A, B and C, respectively. Details of this classification can be found in the original report of ice XV.^94^

**Figure 2:**
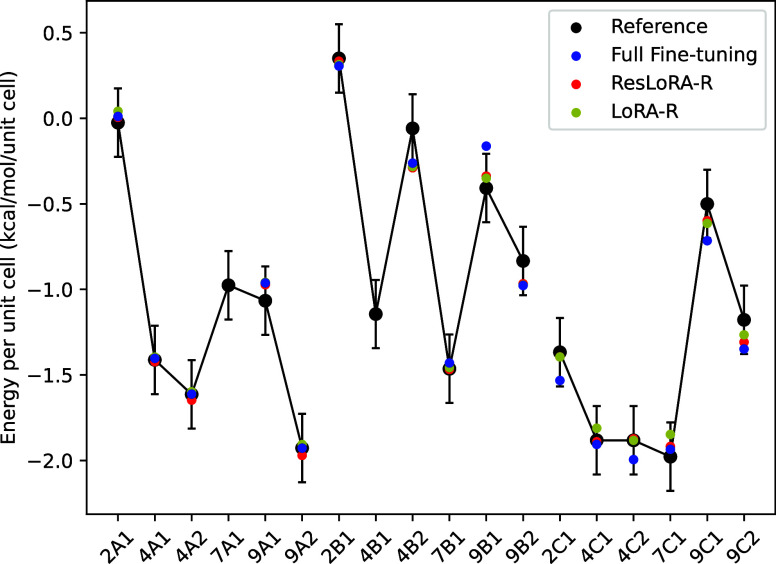
Reference (periodic MP2, this work) and predicted energies for ice-XV supercells (kcal/mol/unit cell) with full-FT, LoRA-R and ResLoRA-R for various hydrogen orderings. The base model is trained on primitive unit cell configurations of the ice XV-4B1, and then fine-tuned with 1 ice XV-4B1 2 × 2 × 2 supercell structure. Predictions on the outlier, i.e., ice XV-7A1 supercell are removed. The caps mark an error boundary of 0.2 kcal/mol/unit cell as visual aid. Best models are presented. See Figure S8 with aligned reference dots for clearer difference between the fine-tuning methods.

**Figure 3:**
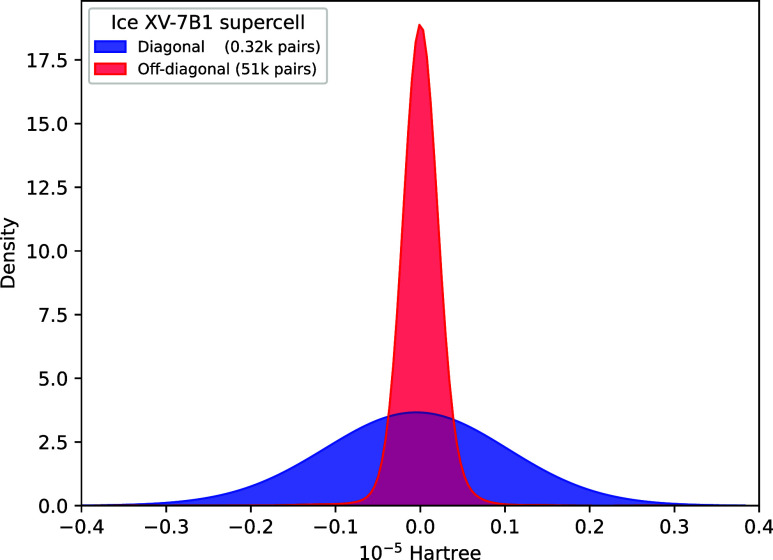
Kernel density estimate for the deviations of predicted orbital pair energies of a single ice-XV 7B1 supercell structure at the optimized geometry. The symmetric distribution around zero, together with a large number of pairs, allows individual pair prediction errors to cancel out in the final summation of total energy, giving error as low as 0.004 kcal/mol per unit cell.

We pretrained the T-dNN base model on the thermalized configurations in the primitive unit cell of the ice XV-4B1 conformation which contains 10 thermally fluctuated water molecules around the equilibrated structure. This makes it interesting to see if it can predict other hydrogen orderings in supercells. The base model predictions on the 2 × 2 × 2 supercells without fine-tuning have a large MAE of 48.8 kcal/mol (6.1 kcal/mol/unit cell). Surprisingly, by retraining only one ice XV-4B1 2 × 2 × 2 supercell at the optimized geometry, the best model predictions on all other ice XV structures are already very accurate (Table S8 in Supporting Information), giving as low as 0.062 kcal/mol MAE per unit cell, and as low as 0.004 kcal/mol deviation per unit cell for the ice XV-7B1 structure. Explicitly training a large number of pairs not only reduces the data requirement but also facilitates error cancellation rather than error accumulation as seen in Figure 3. It is important to note that this can be statistically converged to the mean, i.e, zero error, if the underlying prediction is unbiased. Among the accurate predictions of all models, only ResLoRA-R maintains the reference hydrogen ordering in energetically close region (see ice XV-4C1 to ice XV-7C1 in Figure 2).

It is worth noting that there is a large outlier associated with the ice XV-7A1 structure, with as large as 2.5 kcal/mol deviation per unit cell for all models. The pair energy distribution analysis (Figure S9) in Supporting Information reveals that its reference pair energy distribution is drastically different from all other structures, and therefore the outlier is unrelated to the performance of the model. This suggests that unbiased preparation of both the pretraining and fine-tuning data sets is necessary. It is desirable that the former is used to capture conformational degrees of freedom in the small systems and the latter can solely focus on the system size change upon transitioning to large systems.

### 3.5 Neural Network Analysis

**Figure 4:**
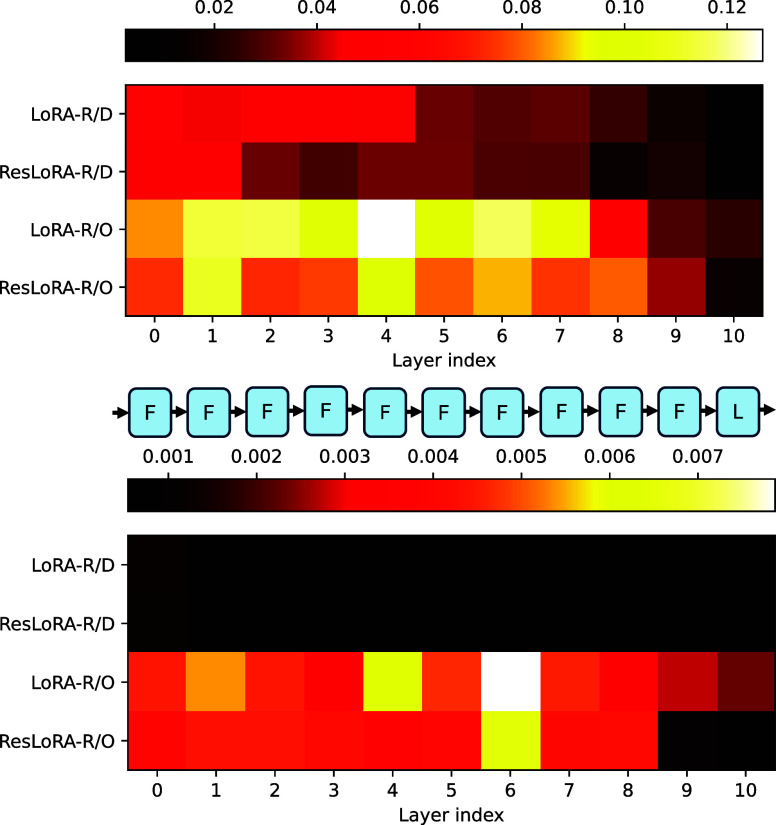
Heat map showing the Frobenius norm ratio ∥Δ**W**∥/∥**W**_0_∥ of various hidden layers arranged in sequential order for fine-tuned models on liquid (H_2_O)_64_ (top) and ice XV hydrogen orderings (bottom). The layer index 10 corresponds to the linear layer that explicitly learns Δ**e**_**ij**_^**R**^. D and O denote diagonal pairs and off-diagonal pairs, respectively. The simplified base model is placed at the center. Note that the ranges of color bars are not the same.

It is advantageous to analyze how the proposed architectures adjust the pretrained model through the hidden layers of the neural network during fine-tuning. We first concern with the Δ**W** of the LoRA blocks, and we report the Frobenius norm ratio of ∥Δ**W**∥/∥**W**_0_∥ to measure how much the original weight matrices have been fine-tuned in various layers when adapting to larger length scales with the same theory and basis set. In Figure 4, the heat map is shown for the liquid system with models fine-tuned on 20 (H_2_O)_64_ (see Figures S10–S18 in Supporting Information for more details on other systems). In general, both LoRA-R and ResLoRA-R make small changes to the original weight matrices, which indicates that the underlying representations are well captured in the smaller systems and require only modest adjustment in the larger systems. The diagonal pairs require less fine-tuning on the weights and it is consistent with the fact that individual orbitals remain transferable, while off-diagonal orbital pairs have extended spatial distributions which differ significantly from small to large. Direct fine-tuning on larger systems with higher level of theory and basis set is not desirable due to the expensive preparation of data set. However, the required data size can be drastically reduced when the most complicated generalization problems (target level of theory, basis set and conformational degrees of freedom) are handled in smaller systems. Similar heat maps are shown in Figure S19 for the protonated water clusters, which are first fine-tuned with smaller protonated clusters. It is apparent that the Frobenius norm ratios are substantially larger (see the ranges of color bars) with respect to the subsequent fine-tuning across length scale as seen in Figures S10, S12 and S14. In passing, it is also observed that fine-tuning the base model across varying theory levels and basis sets is indeed much more difficult than generalizing length scale, as clearly revealed in Figure S19.

It is worth noting that for fine-tuning on larger systems, hidden layers close to the output layer are less perturbed than earlier layers, which is the most apparent for diagonal pairs. The observation remains similar using fewer hidden layers as seen in Figures S20 and S21. The network numerical error propagation is not the cause of the observed weight change, as during the backpropagation the weight update in earlier layers in principle suffers more from gradient vanishing problem than that in the last few hidden layers closer to the output. In fact, it suggests later layers encode common transformations that remain relatively intact, which might be useful to utilize varying LoRA ranks in different layers in an efficient way to further prune down unnecessary parameters with AdaLoRa.^95^ The previous layers can be interpreted as system-dependent layers responsible for absorbing new knowledge obtained from larger systems. The trend across layers is less obvious for off-diagonal pairs which are more sensitive to system size, especially when the pretraining system is small. It is observed that the use of residual connections among the LoRA blocks in ResLoRA-R restricts large Δ**W**, and this might be related to the observed low variance relative to LoRA-R. The latter is more flexible in updating the weights, and this could explain why it is generally better in terms of accuracy of its best models. However, in some challenging cases such as distinguishing energetically close structures in ice XV, ResLoRA-R makes generally robust predictions as seen in Figure 2 and Table S8 in Supporting Information. It is also shown (Figure 4, layer index 10) that the residual connection to learn explicitly Δ**e**_**ij**_^**R**^ indeed learns the small and delicate correction to individual pairs as indicated by its very small weight ratio relative to the final linear layer in the base model that predicts the pair energy **e**_**ij**_^**p**^.

## 4 Conclusions

In this study, we have demonstrated the strength of various neural network architectures in predicting chemically accurate electron correlation energies of large molecular systems with a pool of only several hundred training energies of small molecules. Our main results reveal that a pretraining model on smaller, well-characterized and unbiased systems, when fine-tuned with residual connection and low-rank adaptation techniques, can significantly enhance model performance and transferability. For instances, by fine-tuning the pretrained T-dNN model with 129 CCSD(T)/def2-TZVP energy data for protonated water clusters, ranging from (H_2_O)_5_(H_3_O)^+^ to (H_2_O)_8_(H_3_O)^+^, we achieved the gold-standard CCSD(T)/def2-TZVP energy predictions with MAEs below 0.50 kcal/mol for (H_2_O)_20_(H_3_O)^+^. An extension of the molecular T-dNN model to the periodic one for crystals has been implemented by expressing the pseudo energy feature at Γ-point in the momentum space. The periodic T-dNN base model can be efficiently retrained using various low-rank fine-tuning techniques, as demonstrated in the periodic MP2 energy prediction of liquid water and poly glycine chain at chemical accuracy similar to molecular prediction. These results underscore the model robustness and reliability in accurately capturing and transferring the effects across basis sets, theory levels, and molecular sizes.

Our low-data neural network approach in quantum chemistry demonstrates a potential to overcome the high computational costs in conventional machine learning models which usually demand an exceedingly large number of quantum chemical calculations for training data generation. By leveraging pretraining on small systems, we can efficiently encode fundamental chemical knowledge into the model. Fine-tuning this pretrained model on a limited data set allows us to transfer this knowledge to larger and more complex systems, thereby reducing the need for extensive additional data. Our findings also suggest that the deeper layers of the neural network, which are closer to the output, require less fine-tuning compared to the initial layers near the input. This insight implies that deeper layer parameters transmit more general and transferable knowledge to the output, while the initial layers are more adaptable and system-dependent to unknown systems. In particular, the use of residual connections to T-dNN, which leads to the ResT-dNN model, learns delicate pair energy corrections for individual orbital pairs, and plays a crucial role in directly transmitting the feature pattern to the output for challenging cases.

This study represents an important step forward in the quest for cost-effective and highly accurate and transferable machine learning models in quantum chemistry. Our future work will center on expanding the range of molecular systems and properties that can be predicted using these techniques in quantum chemistry. Significant efforts will be put into implementing novel T-dNN models for predicting and assessing molecular and crystalline geometries that would inspire new methods for fast molecular search in chemical space. Furthermore, it is promising to generalize the idea of the pairwise energy decomposition to MLIPs using a similar architecture to that of Allegro.^56^ By explicitly supervising individual pair contributions with computed reference values, we anticipate a drastic reduction of the data requirement of MLIPs. Finally, an injection of intermediate LMO layers to the architecture from an efficient upstream ML task (e.g., through SchNorb^57^) and the supervision of LMO-pair energy predictions will further unleash the full capacity of the present electron-based and atom-based ML models.

## Supporting Information Available

The Supporting Information contains further results of the computations in the following and is available free of charge. Target systems and illustrative particle-hole excitations; pair energy distributions; prediction errors on Liquid water, (Gly)_*n*_ chain and protonated water clusters; binding curves of charged dimers; prediction errors on ice-XV sublattice; architectural analysis across layers (PDF)

## References

[ref1] Martin J. M. L. Electron Correlation: Nature’s Weird and Wonderful Chemical Glue. Isr. J. Chem. 2022, 62, e202100111 10.1002/ijch.202100111.

[ref2] He X.; Fusti-Molnar L.; Cui G.; Merz K. M. Jr Importance of dispersion and electron correlation in ab initio protein folding. J. Phys. Chem. B 2009, 113, 5290–5300. 10.1021/jp8106952. 19320454 PMC2737261

[ref3] Grimme S. On the Importance of Electron Correlation Effects for the π-π Interactions in Cyclophanes. Chem. -Eur. J. 2004, 10, 3423–3429. 10.1002/chem.200400091. 15252788

[ref4] Hu S. X. Boosting photoabsorption by attosecond control of electron correlation. Phys. Rev. Lett. 2013, 111, 123003 10.1103/PhysRevLett.111.123003. 24093257

[ref5] Tew D. P.; Klopper W.; Helgaker T. Electron correlation: The many-body problem at the heart of chemistry. J. Comput. Chem. 2007, 28, 1307–1320. 10.1002/jcc.20581. 17269126

[ref6] Huang B.; Von Lilienfeld O. A. Ab initio machine learning in chemical compound space. Chem. Rev. 2021, 121, 10001–10036. 10.1021/acs.chemrev.0c01303. 34387476 PMC8391942

[ref7] Aldossary A.; Campos-Gonzalez-Angulo J. A.; Pablo-García S.; Leong S. X.; Rajaonson E. M.; Thiede L.; Tom G.; Wang A.; Avagliano D.; Aspuru-Guzik A. In silico chemical experiments in the Age of AI: From quantum chemistry to machine learning and back. Adv. Mater. 2024, 36, 2402369 10.1002/adma.202402369. 38794859

[ref8] Zhao W. X.; Zhou K.; Li J.; Tang T.; Wang X.; Hou Y.; Min Y.; Zhang B.; Zhang J.; Dong Z.; Du Y.; Yang C.; Chen Y.; Chen Z.; Jiang J.; Ren R.; Li Y.; Tang X.; Liu Z.; Liu P.; Nie J.-Y.; Wen J.-R. A Survey of Large Language Models, 2023, arXiv:2303.18223. arXiv.org e-Print archive https://arxiv.org/abs/2303.18223.

[ref9] Kaplan J.; McCandlish S.; Henighan T.; Brown T. B.; Chess B.; Child R.; Gray S.; Radford A.; Wu J.; Amodei D. Scaling Laws for Neural Language Models. 2020.

[ref10] Villalobos P.; Ho A.; Sevilla J.; Besiroglu T.; Heim L.; Hobbhahn M. Will We Run Out of Data? Limits of LLM Scaling Based on Human-generated Data, 2024, arXiv:2211.04325. arXiv.org e-Print archive https://arxiv.org/abs/2211.04325.

[ref11] Muennighoff N.; Rush A.; Barak B.; Le Scao T.; Tazi N.; Piktus A.; Pyysalo S.; Wolf T.; Raffel C. A. Scaling data-constrained language models. Advances in Neural Information Processing Systems. Adv. Neural Inf. Process. Syst. 2024, 36, 50358.

[ref12] Jain A.; Ong S. P.; Hautier G.; Chen W.; Richards W. D.; Dacek S.; Cholia S.; Gunter D.; Skinner D.; Ceder G.; Persson K. A. Commentary: The Materials Project: A materials genome approach to accelerating materials innovation. APL Mater. 2013, 1, 011002 10.1063/1.4812323.

[ref13] Zhu L.; Zhou J.; Sun Z. Materials data toward machine learning: advances and challenges. J. Phys. Chem. Lett. 2022, 13, 3965–3977. 10.1021/acs.jpclett.2c00576. 35481746

[ref14] Dauparas J.; Anishchenko I.; Bennett N.; Bai H.; Ragotte R. J.; Milles L. F.; Wicky B. I. M.; Courbet A.; de Haas R. J.; Bethel N.; Leung P. J. Y.; Huddy T. F.; Pellock S.; Tischer D.; Chan F.; Koepnick B.; Nguyen H.; Kang A.; Sankaran B.; Bera A. K.; King N. P.; Baker D. Robust deep learning–based protein sequence design using ProteinMPNN. Science 2022, 378, 49–56. 10.1126/science.add2187. 36108050 PMC9997061

[ref15] Huang B.; von Rudorff G. F.; von Lilienfeld O. A. The central role of density functional theory in the AI age. Science 2023, 381, 170–175. 10.1126/science.abn3445. 37440654

[ref16] Ng W.-P.; Liang Q.; Yang J. Low-data deep quantum chemical learning for accurate MP2 and coupled-cluster correlations. J. Chem. Theory Comput. 2023, 19, 5439–5449. 10.1021/acs.jctc.3c00518. 37506400

[ref17] Heinen S.; Khan D.; von Rudorff G. F.; Karandashev K.; Arrieta D. J. A.; Price A. J.; Nandi S.; Bhowmik A.; Hermansson K.; von Lilienfeld O. A. Reducing training data needs with minimal multilevel machine learning (M3L). Mach. Learn.: Sci. Technol. 2024, 5, 025058 10.1088/2632-2153/ad4ae5.

[ref18] Townsend J.; Vogiatzis K. D. Transferable MP2-Based Machine Learning for Accurate Coupled-Cluster Energies. J. Chem. Theory Comput. 2020, 16, 7453–7461. 10.1021/acs.jctc.0c00927. 33138363

[ref19] Cheng L.; Welborn M.; Christensen A. S.; Miller T. F. III A universal density matrix functional from molecular orbital-based machine learning: Transferability across organic molecules. J. Chem. Phys. 2019, 150, 131103 10.1063/1.5088393. 30954042

[ref20] Cheng L.; Kovachki N. B.; Welborn M.; Miller T. F. III Regression clustering for improved accuracy and training costs with molecular-orbital-based machine learning. J. Chem. Theory Comput. 2019, 15, 6668–6677. 10.1021/acs.jctc.9b00884. 31638804

[ref21] Husch T.; Sun J.; Cheng L.; Lee S. J.; Miller T. F. III Improved accuracy and transferability of molecular-orbital-based machine learning: Organics, transition-metal complexes, non-covalent interactions, and transition states. J. Chem. Phys. 2021, 154, 064108 10.1063/5.0032362. 33588560

[ref22] Cheng L.; Sun J.; Miller T. F. I. Accurate Molecular-Orbital-Based Machine Learning Energies via Unsupervised Clustering of Chemical Space. J. Chem. Theory Comput. 2022, 18, 4826–4835. 10.1021/acs.jctc.2c00396. 35858242

[ref23] Qiao Z.; Christensen A. S.; Welborn M.; Manby F. R.; Anandkumar A.; Miller T. F. III Informing geometric deep learning with electronic interactions to accelerate quantum chemistry. Proc. Natl. Acad. Sci. U.S.A. 2022, 119, e2205221119 10.1073/pnas.2205221119. 35901215 PMC9351474

[ref24] Welborn M.; Cheng L.; Miller T. F. III Transferability in machine learning for electronic structure via the molecular orbital basis. J. Chem. Theory Comput. 2018, 14, 4772–4779. 10.1021/acs.jctc.8b00636. 30040892

[ref25] Grisafi A.; Fabrizio A.; Meyer B.; Wilkins D. M.; Corminboeuf C.; Ceriotti M. Transferable machine-learning model of the electron density. ACS Cent. Sci. 2019, 5, 57–64. 10.1021/acscentsci.8b00551. 30693325 PMC6346381

[ref26] Tsubaki M.; Mizoguchi T. Quantum deep descriptor: Physically informed transfer learning from small molecules to polymers. J. Chem. Theory Comput. 2021, 17, 7814–7821. 10.1021/acs.jctc.1c00568. 34846893

[ref27] Ramakrishnan R.; Dral P. O.; Rupp M.; Von Lilienfeld O. A. Quantum chemistry structures and properties of 134 kilo molecules. Sci. Data 2014, 1, 1–7. 10.1038/sdata.2014.22. PMC4322582 25977779

[ref28] Behler J.; Parrinello M. Generalized neural-network representation of high-dimensional potential-energy surfaces. Phys. Rev. Lett. 2007, 98, 146401 10.1103/PhysRevLett.98.146401. 17501293

[ref29] Faber F. A.; Christensen A. S.; Huang B.; Von Lilienfeld O. A. Alchemical and structural distribution based representation for universal quantum machine learning. J. Chem. Phys. 2018, 148, 241717 10.1063/1.5020710. 29960351

[ref30] Christensen A. S.; Bratholm L. A.; Faber F. A.; Anatole von Lilienfeld O. FCHL revisited: Faster and more accurate quantum machine learning. J. Chem. Phys. 2020, 152, 044107 10.1063/1.5126701. 32007071

[ref31] Zaverkin V.; Kästner J. Gaussian moments as physically inspired molecular descriptors for accurate and scalable machine learning potentials. J. Chem. Theory Comput. 2020, 16, 5410–5421. 10.1021/acs.jctc.0c00347. 32672968

[ref32] Bartók A. P.; Kondor R.; Csányi G. On representing chemical environments. Phys. Rev. B 2013, 87, 184115 10.1103/PhysRevB.87.184115.

[ref33] Thompson A. P.; Swiler L. P.; Trott C. R.; Foiles S. M.; Tucker G. J. Spectral neighbor analysis method for automated generation of quantum-accurate interatomic potentials. J. Comput. Phys. 2015, 285, 316–330. 10.1016/j.jcp.2014.12.018.

[ref34] Smith J. S.; Isayev O.; Roitberg A. E. ANI-1: an extensible neural network potential with DFT accuracy at force field computational cost. Chem. Sci. 2017, 8, 3192–3203. 10.1039/C6SC05720A. 28507695 PMC5414547

[ref35] Zhang L.; Han J.; Wang H.; Car R.; Weinan E. Deep potential molecular dynamics: a scalable model with the accuracy of quantum mechanics. Phys. Rev. Lett. 2018, 120, 143001 10.1103/PhysRevLett.120.143001. 29694129

[ref36] Gilmer J.; Schoenholz S. S.; Riley P. F.; Vinyals O.; Dahl G. E. Neural message passing for quantum chemistry. In International Conference on Machine Learning 2017; p 12631272.

[ref37] Schütt K. T.; Sauceda H. E.; Kindermans P.-J.; Tkatchenko A.; Müller K.-R. Schnet–a deep learning architecture for molecules and materials. J. Chem. Phys. 2018, 148, 241722 10.1063/1.5019779. 29960322

[ref38] Unke O. T.; Meuwly M. PhysNet: A neural network for predicting energies, forces, dipole moments, and partial charges. J. Chem. Theory Comput. 2019, 15, 3678–3693. 10.1021/acs.jctc.9b00181. 31042390

[ref39] Gasteiger J.; Groß J.; Günnemann S. Directional Message Passing for Molecular Graphs. In International Conference on Learning Representations, 2020.

[ref40] Schütt K.; Unke O.; Gastegger M. Equivariant message passing for the prediction of tensorial properties and molecular spectra. International Conference on Machine Learning. 2021, 9377–9388.

[ref41] Batzner S.; Musaelian A.; Sun L.; Geiger M.; Mailoa J. P.; Kornbluth M.; Molinari N.; Smidt T. E.; Kozinsky B. E (3)-equivariant graph neural networks for data-efficient and accurate interatomic potentials. Nat. Commun. 2022, 13, 2453 10.1038/s41467-022-29939-5. 35508450 PMC9068614

[ref42] Deng B.; Zhong P.; Jun K.; Riebesell J.; Han K.; Bartel C. J.; Ceder G. CHGNet as a pretrained universal neural network potential for charge-informed atomistic modelling. Nat. Mach. Intell. 2023, 5, 1031–1041. 10.1038/s42256-023-00716-3.

[ref43] Dick S.; Fernandez-Serra M. Machine learning accurate exchange and correlation functionals of the electronic density. Nat. Commun. 2020, 11, 3509 10.1038/s41467-020-17265-7. 32665540 PMC7360771

[ref44] Chen Y.; Zhang L.; Wang H.; E W. DeePKS: A comprehensive data-driven approach toward chemically accurate density functional theory. J. Chem. Theory Comput. 2021, 17, 170–181. 10.1021/acs.jctc.0c00872. 33296197

[ref45] Li W.; Ou Q.; Chen Y.; Cao Y.; Liu R.; Zhang C.; Zheng D.; Cai C.; Wu X.; Wang H.; et al. DeePKS+ ABACUS as a Bridge between Expensive Quantum Mechanical Models and Machine Learning Potentials. J. Phys. Chem. A 2022, 126, 9154–9164. 10.1021/acs.jpca.2c05000. 36455227

[ref46] Chmiela S.; Tkatchenko A.; Sauceda H. E.; Poltavsky I.; Schütt K. T.; Müller K.-R. Machine learning of accurate energy-conserving molecular force fields. Sci. Adv. 2017, 3, e1603015 10.1126/sciadv.1603015. 28508076 PMC5419702

[ref47] Chmiela S.; Sauceda H. E.; Müller K.-R.; Tkatchenko A. Towards exact molecular dynamics simulations with machine-learned force fields. Nat. Commun. 2018, 9, 3887 10.1038/s41467-018-06169-2. 30250077 PMC6155327

[ref48] Yoo D.; Lee K.; Jeong W.; Lee D.; Watanabe S.; Han S. Atomic energy mapping of neural network potential. Phys. Rev. Mater. 2019, 3, 093802 10.1103/PhysRevMaterials.3.093802.

[ref49] Eriksen J. J. Mean-field density matrix decompositions. J. Chem. Phys. 2020, 153, 214109 10.1063/5.0030764. 33291929

[ref50] Kjeldal F. Ø.; Eriksen J. J. Decomposing chemical space: Applications to the machine learning of atomic energies. J. Chem. Theory Comput. 2023, 19, 2029–2038. 10.1021/acs.jctc.2c01290. 36926874

[ref51] Zamok L.; Eriksen J. J. Atomic Decompositions of Periodic Electronic-Structure Simulations. 2024, arXiv:2407.10148. arXiv.org e-Print archive https://arxiv.org/abs/2407.10148. 10.1021/acs.jpca.4c06651 39719057

[ref52] Rupp M.; Tkatchenko A.; Müller K.-R.; Von Lilienfeld O. A. Fast and accurate modeling of molecular atomization energies with machine learning. Phys. Rev. Lett. 2012, 108, 058301 10.1103/PhysRevLett.108.058301. 22400967

[ref53] Hansen K.; Biegler F.; Ramakrishnan R.; Pronobis W.; Von Lilienfeld O. A.; Muller K.-R.; Tkatchenko A. Machine learning predictions of molecular properties: Accurate many-body potentials and nonlocality in chemical space. J. Phys. Chem. Lett. 2015, 6, 2326–2331. 10.1021/acs.jpclett.5b00831. 26113956 PMC4476293

[ref54] Yao K.; Herr J. E.; Brown S. N.; Parkhill J. Intrinsic bond energies from a bonds-in-molecules neural network. J. Phys. Chem. Lett. 2017, 8, 2689–2694. 10.1021/acs.jpclett.7b01072. 28573865

[ref55] Glick Z. L.; Metcalf D. P.; Koutsoukas A.; Spronk S. A.; Cheney D. L.; Sherrill C. D. AP-Net: An atomic-pairwise neural network for smooth and transferable interaction potentials. J. Chem. Phys. 2020, 153, 044112 10.1063/5.0011521. 32752707

[ref56] Musaelian A.; Batzner S.; Johansson A.; Sun L.; Owen C. J.; Kornbluth M.; Kozinsky B. Learning local equivariant representations for large-scale atomistic dynamics. Nat. Commun. 2023, 14, 579 10.1038/s41467-023-36329-y. 36737620 PMC9898554

[ref57] Schütt K. T.; Gastegger M.; Tkatchenko A.; Müller K.-R.; Maurer R. J. Unifying machine learning and quantum chemistry with a deep neural network for molecular wavefunctions. Nat. Commun. 2019, 10, 5024 10.1038/s41467-019-12875-2. 31729373 PMC6858523

[ref58] Unke O.; Bogojeski M.; Gastegger M.; Geiger M.; Smidt T.; Müller K.-R. SE (3)-equivariant prediction of molecular wavefunctions and electronic densities. Adv. Neural Inf. Process. Syst. 2021, 34, 14434–14447.

[ref59] Nigam J.; Willatt M. J.; Ceriotti M. Equivariant representations for molecular Hamiltonians and N-center atomic-scale properties J. Chem. Phys. 2022; Vol. 156 10.1063/5.0072784. 34998321

[ref60] Gong X.; Li H.; Zou N.; Xu R.; Duan W.; Xu Y. General framework for E (3)-equivariant neural network representation of density functional theory Hamiltonian. Nat. Commun. 2023, 14, 2848 10.1038/s41467-023-38468-8. 37208320 PMC10199065

[ref61] Westermayr J.; Maurer R. J. Physically inspired deep learning of molecular excitations and photoemission spectra. Chem. Sci. 2021, 12, 10755–10764. 10.1039/D1SC01542G. 34447563 PMC8372319

[ref62] Cignoni E.; Suman D.; Nigam J.; Cupellini L.; Mennucci B.; Ceriotti M. Electronic Excited States from Physically Constrained Machine Learning. ACS Cent. Sci. 2024, 10, 637–648. 10.1021/acscentsci.3c01480. 38559300 PMC10979507

[ref63] Tang H.; Xiao B.; He W.; Subasic P.; Harutyunyan A. R.; Wang Y.; Liu F.; Xu H.; Li J. Multi-task learning for molecular electronic structure approaching coupled-cluster accuracy. arXiv preprint arXiv:2405.12229 2024, arXiv:2405.12229. arXiv.org e-Print archive https://arxiv.org/abs/2405.12229.

[ref64] He K.; Zhang X.; Ren S.; Sun J. Deep residual learning for image recognition. In Proceedings of the IEEE conference on computer vision and pattern recognition. 2016; pp 770–778.

[ref65] Hu E. J.; Shen Y.; Wallis P.; Allen-Zhu Z.; Li Y.; Wang S.; Wang L.; Chen W. LoRA: Low-Rank Adaptation of Large Language Models. The International Conference on Learning Representations, ICLR 2022, Virtual Event, April 25–29, 2022. 2022..

[ref66] Ben Zaken E.; Goldberg Y.; Ravfogel S. BitFit: Simple Parameter-efficient Fine-tuning for Transformer-based Masked Language-models Proceedings of the Annual Meeting of the Association for Computational Linguistics (Volume 2: Short Papers), Dublin, Ireland, 2022, pp 1–9.

[ref67] Yang J.; Kurashige Y.; Manby F. R.; Chan G. K. Tensor factorizations of local second-order Møller-Plesset theory. J. Chem. Phys. 2011, 134, 044123 10.1063/1.3528935. 21280703

[ref68] Pulay P.; Saebø S. Orbital-invariant formulation and second-order gradient evaluation in Møller-Plesset perturbation theory. Theor. Chim. Acta 1986, 69, 357–368. 10.1007/BF00526697.

[ref69] Yang J. Making quantum chemistry compressive and expressive: Toward practical ab-initio simulation. WIREs Comput Mol Sci. 2024, 14, e1706 10.1002/wcms.1706.

[ref70] Shi S.; Huang S.; Song M.; Li Z.; Zhang Z.; Huang H.; Wei F.; Deng W.; Sun F.; Zhang Q. ResLoRA: Identity Residual Mapping in Low-Rank Adaption. In Findings of the Association for Computational Linguistics ACL 2024. Bangkok, 2024; pp 8870–8884.

[ref71] Cooper A. M.; Kästner J.; Urban A.; Artrith N. Efficient training of ANN potentials by including atomic forces via Taylor expansion and application to water and a transition-metal oxide. npj Comput. Mater. 2020, 6, 54 10.1038/s41524-020-0323-8.

[ref72] Wales D. J.; Doye J. P. K.; Dullweber A.; Hodges M. P.; Naumkin F. Y.; Calvo F.; Hernández-Rojas J.; Middleton T. F. The Cambridge Cluster Database. http://www-wales.ch.cam.ac.uk/CCD.html.

[ref73] Hodges M. P.; Wales D. J. Global minima of protonated water clusters. Chem. Phys. Lett. 2000, 324, 279–288. 10.1016/S0009-2614(00)00584-4.

[ref74] Del Ben M.; VandeVondele J.; Slater B. Periodic MP2, RPA, and boundary condition assessment of hydrogen ordering in ice XV. J. Phys. Chem. Lett. 2014, 5, 4122–4128. 10.1021/jz501985w. 26278943

[ref75] Kühne T. D.; Iannuzzi M.; Del Ben M.; Rybkin V. V.; Seewald P.; Stein F.; Laino T.; Khaliullin R. Z.; Schuett O.; Schiffmann F.; Golze D.; Wilhelm J.; Chulkov S.; Bani-Hashemian M. H.; Weber V.; Borstnik U.; Taillefumier M.; Jakobovits A. S.; Lazzaro A.; Pabst H.; Mueller T.; Schade R.; Guidon M.; Andermatt S.; Holmberg N.; Schenter G. K.; Hehn A.; Bussy A.; Belleflamme F.; Tabacchi G.; Gloess A.; Lass M.; Bethune I.; Mundy C. J.; Plessl C.; Watkins M.; VandeVondele J.; Krack M.; Hutter J. CP2K: An electronic structure and molecular dynamics software package - Quickstep: Efficient and accurate electronic structure calculations. J. Chem. Phys. 2020, 152, 194103 10.1063/5.0007045. 33687235

[ref76] Sun Q.; Zhang X.; Banerjee S.; Bao P.; Barbry M.; Blunt N. S.; Bogdanov N. A.; Booth G. H.; Chen J.; Cui Z.-H.; Eriksen J. J.; Gao Y.; Guo S.; Hermann J.; Hermes M. R.; Koh K.; Koval P.; Lehtola S.; Li Z.; Liu J.; Mardirossian N.; McClain J. D.; Motta M.; Mussard B.; Pham H. Q.; Pulkin A.; Purwanto W.; Robinson P. J.; Ronca E.; Sayfutyarova E. R.; Scheurer M.; Schurkus H. F.; Smith J. E. T.; Sun C.; Sun S.-N.; Upadhyay S.; Wagner L. K.; Wang X.; White A.; Whitfield J. D.; Williamson M. J.; Wouters S.; Yang J.; Yu J. M.; Zhu T.; Berkelbach T. C.; Sharma S.; Sokolov A. Y.; Chan G. K.-L. Recent developments in the PySCF program package. J. Chem. Phys. 2020, 153, 024109 10.1063/5.0006074. 32668948

[ref77] Lehtola S.; Jónsson H. Pipek–Mezey orbital localization using various partial charge estimates. J. Chem. Theory Comput. 2014, 10, 642–649. 10.1021/ct401016x. 26580041

[ref78] Liang Q.; Yang J. Third-Order Many-Body Expansion of OSV-MP2 Wave Function for Low-Order Scaling Analytical Gradient Computation. J. Chem. Theory Comput. 2021, 17, 6841–6860. 10.1021/acs.jctc.1c00581. 34704757

[ref79] Zhou R.; Liang Q.; Yang J. Complete osv-mp2 analytical gradient theory for molecular structure and dynamics simulations. J. Chem. Theory Comput. 2020, 16, 196–210. 10.1021/acs.jctc.9b00806. 31815490

[ref80] Neese F. Software update: the ORCA program system, version 5.0. WIREs Comput. Mol. Sci. 2022, 12, e1606 10.1002/wcms.1606.

[ref81] Pedregosa F.; Varoquaux G.; Gramfort A.; Michel V.; Thirion B.; Grisel O.; Blondel M.; Prettenhofer P.; Weiss R.; Dubourg V.; Vanderplas J.; Passos A.; Cournapeau D.; Brucher M.; Perrot M.; Duchesnay E. Scikit-learn: Machine Learning in Python. J. Mach. Learn. Res. 2011, 12, 2825–2830.

[ref82] Agarap A. F. Deep Learning using Rectified Linear Units (ReLU). 2019, arXiv:1803.08375. arXiv.org e-Print archive https://arxiv.org/abs/1803.08375.

[ref83] Kingma D. P.; Ba J. Adam: A Method for Stochastic Optimization, 2017, arXiv:1412.6980. arXiv.org e-Print archive https://arxiv.org/abs/1412.6980.

[ref84] He K.; Zhang X.; Ren S.; Sun J. Delving deep into rectifiers: Surpassing human-level performance on imagenet classification. In Proceedings of the IEEE International Conference on Computer Vision, 2015; pp 1026–1034.

[ref85] Chollet F. K. Github, San Francisco, CA, 2015.

[ref86] Chen M. S.; Lee J.; Ye H.-Z.; Berkelbach T. C.; Reichman D. R.; Markland T. E. Data-efficient machine learning potentials from transfer learning of periodic correlated electronic structure methods: Liquid water at AFQMC, CCSD, and CCSD (T) accuracy. J. Chem. Theory Comput. 2023, 19, 4510–4519. 10.1021/acs.jctc.2c01203. 36730728

[ref87] López-Zorrilla J.; Aretxabaleta X. M.; Manzano H. Exploring the polymorphism of dicalcium silicates using transfer learning enhanced machine learning atomic potentials. J. Chem. Theory Comput. 2024, 20, 7682–7690. 10.1021/acs.jctc.4c00479. 39171744

[ref88] Burns L. A.; Faver J. C.; Zheng Z.; Marshall M. S.; Smith D. G.; Vanommeslaeghe K.; MacKerell A. D. Jr; Merz K. M. Jr; Sherrill C. D. The BioFragment Database (BFDb): An open-data platform for computational chemistry analysis of noncovalent interactions. J. Chem. Phys. 2017, 147, 161727 10.1063/1.5001028. 29096505 PMC5656042

[ref89] Grisafi A.; Nigam J.; Ceriotti M. Multi-scale approach for the prediction of atomic scale properties. Chem. Sci. 2021, 12, 2078–2090. 10.1039/D0SC04934D. PMC8179303 34163971

[ref90] Huguenin-Dumittan K. K.; Loche P.; Haoran N.; Ceriotti M. Physics-inspired equivariant descriptors of nonbonded interactions. J. Phys. Chem. Lett. 2023, 14, 9612–9618. 10.1021/acs.jpclett.3c02375. 37862712 PMC10626632

[ref91] Hansen T. C. The everlasting hunt for new ice phases. Nat. Commun. 2021, 12, 3161 10.1038/s41467-021-23403-6. 34039991 PMC8154907

[ref92] Yang J.; Hu W.; Usvyat D.; Matthews D.; Schütz M.; Chan G. K.-L. Ab initio determination of the crystalline benzene lattice energy to sub-kilojoule/mol accuracy. Science 2014, 345, 640–643. 10.1126/science.1254419. 25104379

[ref93] Salzmann C. G.; Radaelli P. G.; Slater B.; Finney J. L. The polymorphism of ice: five unresolved questions. Phys. Chem. Chem. Phys. 2011, 13, 18468–18480. 10.1039/c1cp21712g. 21946782

[ref94] Salzmann C. G.; Radaelli P. G.; Mayer E.; Finney J. L. Ice XV: a new thermodynamically stable phase of ice. Phys. Rev. Lett. 2009, 103, 105701 10.1103/PhysRevLett.103.105701. 19792330

[ref95] Zhang Q.; Chen M.; Bukharin A.; He P.; Cheng Y.; Chen W.; Zhao T. Adaptive Budget Allocation for Parameter-Efficient Fine-Tuning. In The Eleventh International Conference on Learning Representations, 2023.

