# Supplementary material for: Accurate Neural Network Fine-Tuning Approach for Transferable Ab Initio Energy Prediction across Varying Molecular and Crystalline Scales
Source: J Chem Theory Comput. 2025 Feb 4;21(4):1602–14. doi: 10.1021/acs.jctc.4c01261 (PMC11866754; doi:10.1021/acs.jctc.4c01261)
Supplement: Supplementary file 1 — ct4c01261_si_001.pdf [file ct4c01261_si_001.pdf]

Supporting Information: Accurate Neural Network Fine-tuning Approach for Transferable *Ab-Initio* Energy Prediction Across Varying Molecular and Crystalline Scales

Wai-Pan Ng[†,‡] Zili Zhang[†,‡] and Jun Yang[*,‡,¶]

‡*Department of Chemistry, The University of Hong Kong, Hong Kong, 999077, P.R. China*

¶*Hong Kong Quantum AI Lab Limited, Hong Kong, 999077, P.R. China*

E-mail: juny@hku.hk

[†]W.-P. N. and Z. Z. contributed equally to this study.

Target systems and illustrative particle-hole excitations

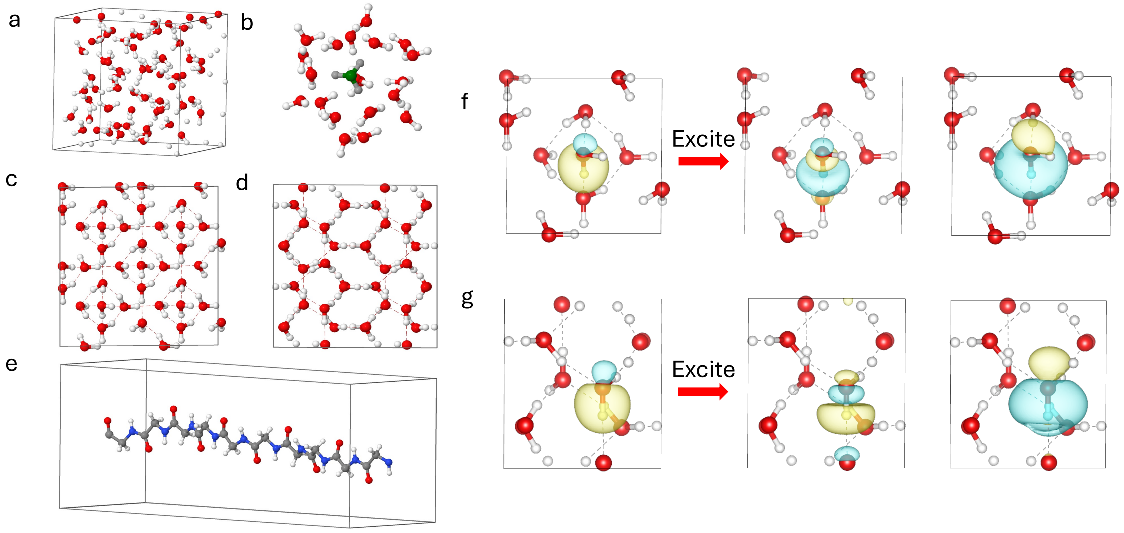

Figure S1: Left: Target systems. (a) Liquid $(H_2O)_{64}$. (b) Protonated water clusters $(H_2O)_{20}(H_3O)^+$. (c-d) Ice XV structures (7A1, 4B1) (e) 1-D $(Gly)_{10}$. Right: Illustrative particle-hole excitations from a LMO with Pipek-Mezey localization to OSVs. (f) Ice XV-7A1. (g) Ice XV-4B1.

Pair energy distributions

Diving deeper into the expression of E_{HF} in terms of one particle terms (h), which is the sum of kinetic energy and nucleus-electron attraction, and two particle Coulomb repulsion (J) and exchange interaction (K) terms,

$$E_{HF} = 2\sum_{i} h_{ii} + \sum_{ij} 2[ii|jj] - [ij|ij] \tag{1}$$

$$= 2\sum_{i} h_{ii} + \sum_{ij} 2J_{ij} - K_{ij} \tag{2}$$

$$= \sum_{i} h_{ii} + \epsilon_{ii} \tag{3}$$

where ϵ_{ii} is the more familiar orbital energy from a SCF calculation and the occupied orbital pairs only appear in the intermediate steps. One can define,

$$e_{ii}^{\text{tot}} = e_{ii} + 2h_{ii} + J_{ii} \tag{4}$$

$$e_{ij}^{\text{tot}} = e_{ij} + 2J_{ij} - K_{ij} \tag{5}$$

for diagonal and off-diagonal pairs, respectively. The diagonal pair contains the one-particle contribution (h_{ii}) from both electron kinetic energy and nucleus-electron attraction energy, as well as the intra-pair Coulomb repulsion (J_{ii}), while the off-diagonal pair contains the pairwise Coulomb (J_{ij}) and exchange (K_{ij}) interactions. The correlated contribution e_{ij} can be thought of as having the correction to mean-field pairs by accounting for explicit electron-electron correlations. Therefore, the total electronic energy is simply written in terms of orbital pairs,

$$E_{\text{tot}} = \sum_{ij} e_{ij}^{\text{tot}} \tag{6}$$

We have calculated a distribution of the total pair energies as defined above for simple ethane and isobutane molecules at CCSD(T)/def2-TZVP level of theory for all electrons. As seen in

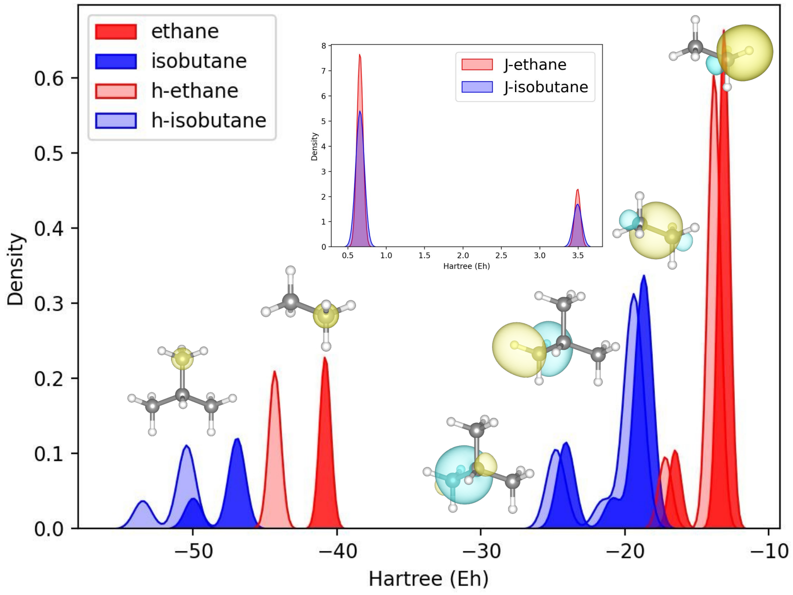

Figure S2: Kernel density estimate for the orbital pairwise decomposition of total electronic energies of ethane and isobutane. Diagonal pair energies $e_{ii}^{tot} = e_{ii} + 2h_{ii} + J_{ii}$ are shown. For off-diagonal pairs and correlation energy alone decomposition, see Figures S3-5. Prefix h omits J_{ii} and retains $2h_{ii}$ in the summation, prefix J omits $2h_{ii}$ and retains J_{ii} and e_{ii}.

Figure S2, the density distributions of the electron-electron pair interaction energies (J-prefixed, including classical Coulomb and correlation energies) are similarly positioned and largely superimposed between ethane and isobutane molecules, while the one particle energy distribution (h-prefixed, referring to the kinetic energy and external electron-nucleus interactions) on the diagonal pairs, determines the overall differences of the landscape of total pair energies between different alkanes. This is well understood in the DFT framework: the $e_{ij} + 2J_{ij} - K_{ij}$ is a reminiscent of the universal electron-electron interactions and the external interactions out of h_{ii} are strongly system-dependent but inexpensive to incorporate as prior knowledge. A striking difference is that the distribution as shown in Figure S2 moves beyond the one particle picture in DFT by explicitly considering the pairwise interactions. This opens up a new possibility for learning a total energy functional in terms of LMO pairs, treating them as fundamental variables instead of the electron density, for which the existing DFT functionals lack transferability.

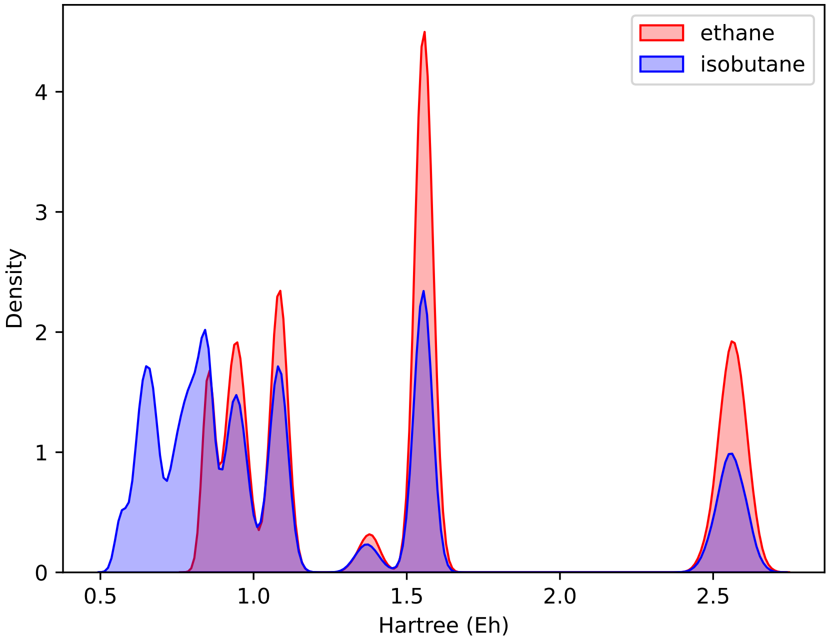

Figure S3: Kernel density estimate for the orbital pairwise decomposition of total electronic energies of ethane and isobutane. Off-diagonal pair energies $e_{ij}^{tot} = e_{ij} + 2J_{ij} - K_{ij}$ are shown.

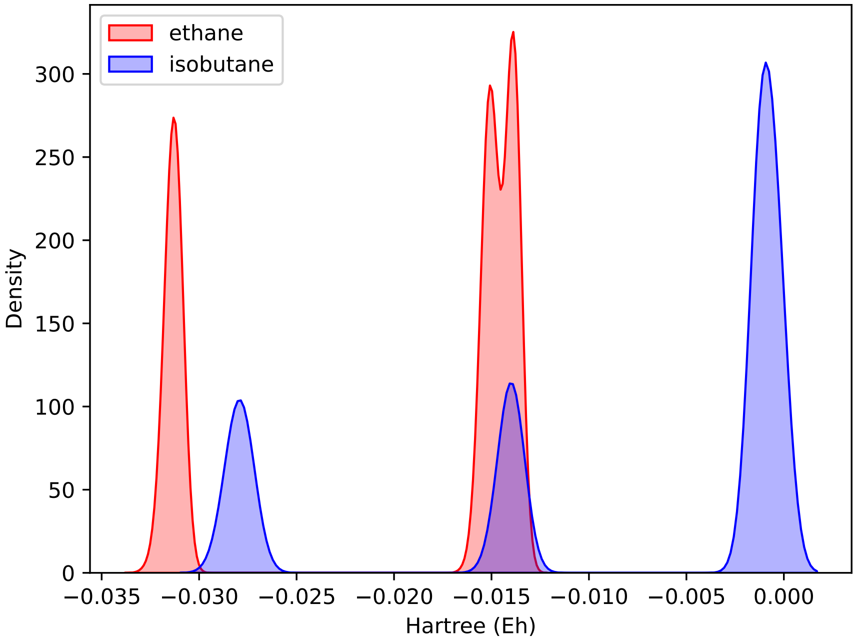

Figure S4: Kernel density estimate for the orbital pairwise decomposition of correlation energies of ethane and isobutane. Diagonal pair energies e_{ii} are shown.

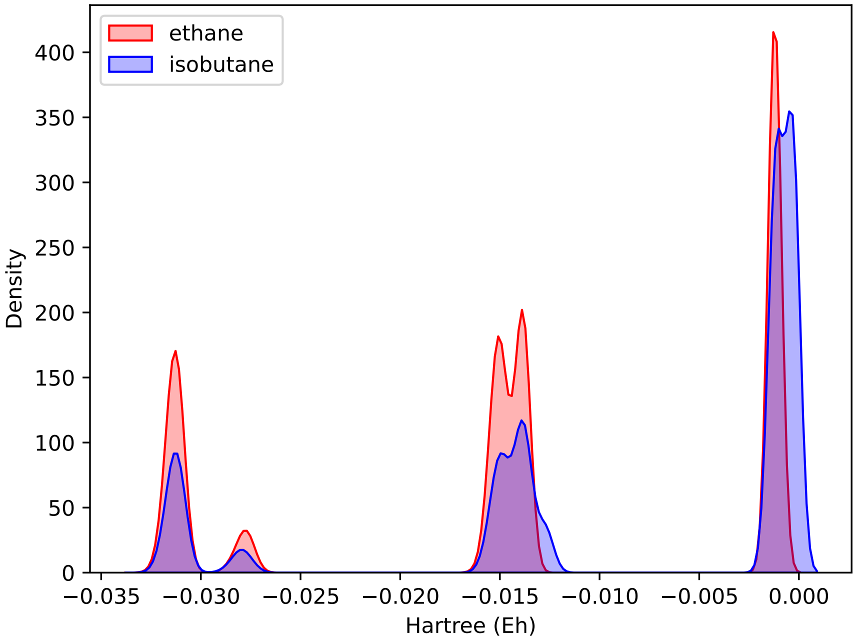

Figure S5: Kernel density estimate for the orbital pairwise decomposition of correlation energies of ethane and isobutane. Off-diagonal pair energies e_{ij} are shown.

Periodic systems: Liquid water and (Gly)n chain

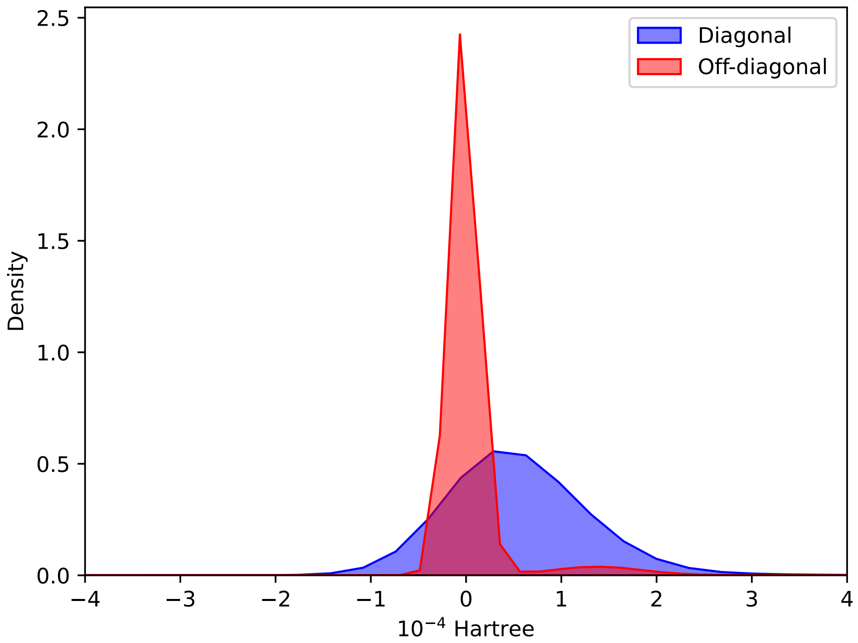

Figure S6: Kernel density estimate for deviations of individual pair energy predictions on liquid water $(H_2O)_{64}$ with a base model pretrained on small $(H_2O)_6$ clusters in periodic boxes, which has 33.8 kcal/mol MAE. It is apparent that individual pair energy errors are actually much smaller, and also the error distributions are biased.

Table S1: Prediction MAE (kcal/mol) on liquid water $(H_2O)_{64}$ with different numbers of fine-tuning molecules with a base model pretrained on small $(H_2O)_6$ clusters in periodic boxes. Base model predictions result in MAE of 33.8 kcal/mol. Results for various fine-tuning approaches are shown as mean±std(best) over six model repetitions.

FT no.	BitFit	BitFit-R	LoRA-R	ResLoRA-R	Full-FT
1	2.102 ± 0.530	1.914 ± 0.753	1.229 ± 0.250	1.292 ± 0.574	1.257 ± 0.121
	(1.491)	(1.359)	(0.928)	(0.980)	(1.103)
10	1.880 ± 0.817	1.499 ± 0.504	1.228 ± 0.463	1.125 ± 0.245	0.918 ± 0.190
	(1.012)	(1.001)	(0.798)	(0.867)	(0.804)
20	1.427 ± 0.439	1.353 ± 0.599	1.176 ± 0.599	0.728 ± 0.087	0.875 ± 0.173
	(1.021)	(0.942)	(0.706)	(0.669)	(0.691)

Table S2: Prediction MAE (kcal/mol) on liquid water $(H_2O)_{64}$ with different numbers of training molecules with Δ-ML. The base model is trained on small $(H_2O)_6$ clusters in periodic boxes with MAE of 33.8 kcal/mol. Results are shown as mean±std(best) over six model repetitions.

Train no.	Δ-ML
1	1.634 ± 0.776
	(0.985)
10	1.529 ± 0.628
	(0.831)
20	1.015 ± 0.201
	(0.757)

Table S3: Prediction MAE (kcal/mol) on liquid water $(H_2O)_{64}$ with different numbers of training molecules with full fine-tuning using different numbers of OSVs for feature construction. The base models built from 4, 6 and 8 OSVs have MAEs of 31.8, 34.0 and 33.8 kcal/mol, respectively. Results for the best models are shown.

FT no./OSV no.	4	6	8
1	0.944	0.920	1.103
10	0.832	0.825	0.804
20	0.931	0.677	0.691

Table S4: Prediction MAE (kcal/mol) on $(Gly)_{10}$ at GTH-TZVP basis set with a single fine-tuning molecule. The base model is pretrained on $(Gly)_4$ at GTH-DZVP basis set. Base model predictions result in MAE of 27.2 kcal/mol, while a model first fine-tuned on $(Gly)_4$ at GTH-TZVP basis set results in MAE of 0.914 kcal/mol. Results for various fine-tuning approaches are shown as mean±std(best) over six model repetitions.

FT no.	BitFit	BitFit-R	LoRA-R	ResLoRA-R	Full-FT
1	0.535 ± 0.006	0.540 ± 0.002	0.535 ± 0.004	0.549 ± 0.013	0.684 ± 0.083
	(0.528)	(0.538)	(0.530)	(0.536)	(0.550)

Protonated water clusters

Table S5: Prediction MAE (kcal/mol) on protonated clusters $(H_2O)_{20}(H_3O)^+$ at cc-pVTZ basis set with a single fine-tuning molecule. The base model is pretrained on small $(H_2O)_6$ clusters in periodic boxes at GTH-DZVP basis set. Base model predictions result in MAE of 563 kcal/mol on $(H_2O)_{20}(H_3O)^+$, while a model first fine-tuned on smaller $(H_2O)_n(H_3O)^+$, with n=5-8, results in MAE of 2.964 kcal/mol. Results for various fine-tuning approaches are shown as mean±std(best) over six model repetitions.

FT no.	BitFit	BitFit-R	LoRA-R	ResLoRA-R	Full-FT
1	0.463 ± 0.065 (0.399)	0.513 ± 0.101 (0.411)	0.443 ± 0.095 (0.374)	0.404 ± 0.031 (0.373)	0.396 ± 0.020 (0.373)

Table S6: Prediction MAE (kcal/mol) on protonated clusters $(H_2O)_{20}(H_3O)^+$ at cc-pVQZ basis set with a single fine-tuning molecule. The base model is pretrained on small $(H_2O)_6$ clusters in periodic boxes at GTH-DZVP basis set. Base model predictions result in MAE of 837 kcal/mol on $(H_2O)_{20}(H_3O)^+$, while a model first fine-tuned on smaller $(H_2O)_n(H_3O)^+$, with n=5-8, results in MAE of 1.585 kcal/mol. Results for various fine-tuning approaches are shown as mean±std(best) over six model repetitions.

FT no.	BitFit	BitFit-R	LoRA-R	ResLoRA-R	Full-FT
1	0.628 ± 0.030 (0.589)	0.613 ± 0.015 (0.599)	0.623 ± 0.032 (0.596)	0.632 ± 0.016 (0.604)	0.670 ± 0.058 (0.605)

Table S7: Prediction MAE (kcal/mol) on protonated clusters $(H_2O)_{20}(H_3O)^+$ at CCSD(T)/def2-TZVP level of theory with a single fine-tuning molecule. The base model is pretrained on small $(H_2O)_6$ clusters in periodic boxes at periodic MP2/GTH-DZVP level of theory. Base model predictions result in MAE of 688 kcal/mol on $(H_2O)_{20}(H_3O)^+$, while a model first fine-tuned on smaller $(H_2O)_n(H_3O)^+$, with n=5-8, results in MAE of 1.215 kcal/mol. Results for various fine-tuning approaches are shown as mean±std(best) over six model repetitions.

FT no.	BitFit	BitFit-R	LoRA-R	ResLoRA-R	Full-FT
1	0.482 ± 0.050 (0.439)	0.449 ± 0.040 (0.390)	0.417 ± 0.031 (0.370)	0.408 ± 0.039 (0.369)	0.482 ± 0.099 (0.385)

Binding curves of charged dimers

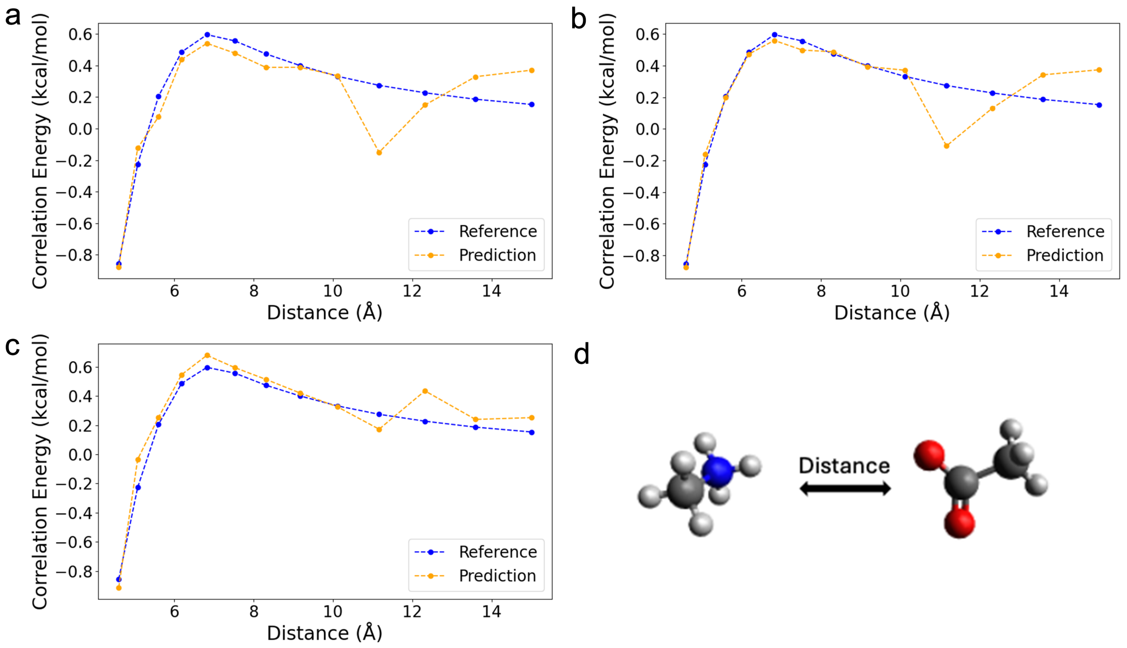

Figure S7: MP2/cc-pVTZ binding curves of a charged-charged dimer $CH_3NH_3^+$ + CH_3COO^-, only correlation energy contribution is shown. The base model is trained on 745 charged-charged dimers from the BFdB database with distances shorter than 7 Å, and tested for approximately 1440 charged-charged dimers at longer distances with the same level of theory and basis set. The base model predictions give MAEs of 0.22 and 0.35 kcal/mol for short and long distances across various dimers, respectively. (a) Binding curve prediction by the base model on the illustrative dimer, 0.13 kcal/mol prediction MAE is obtained for longer distances. (b) Full fine-tuning on short distances, i.e. {4.60, 5.08, 5.60, 6.18, 6.82}. (c) Full fine-tuning on more evenly sampled distances, i.e. {5.08,6.82,9.17,12.32,15.00}. (d) Depiction of the illustrative charged-charged dimer.

Ice XV sublattices

Table S8: Prediction MAE (kcal/mol/unit cell) on the rest of 18 symmetry distinct supercell structures with different hydrogen orderings, with a base model pretrained on ice XV-4B1 primitive unit cells and fine-tuned with 1 ice XV-4B1 2×2×2 structure. Base model predictions result in MAE of 48.8 kcal/mol (6.1 kcal/mol/unit cell). The outlier, i.e. ice XV-7A1 2×2×2 structure, is not included in the MAE. Results for various fine-tuning approaches are shown as mean±std(best) over six model repetitions.

FT no.	LoRA-R	ResLoRA-R	Full-FT
1	0.109 ± 0.033 (0.070)	0.076 ± 0.020 (0.062)	0.101 ± 0.002 (0.097)

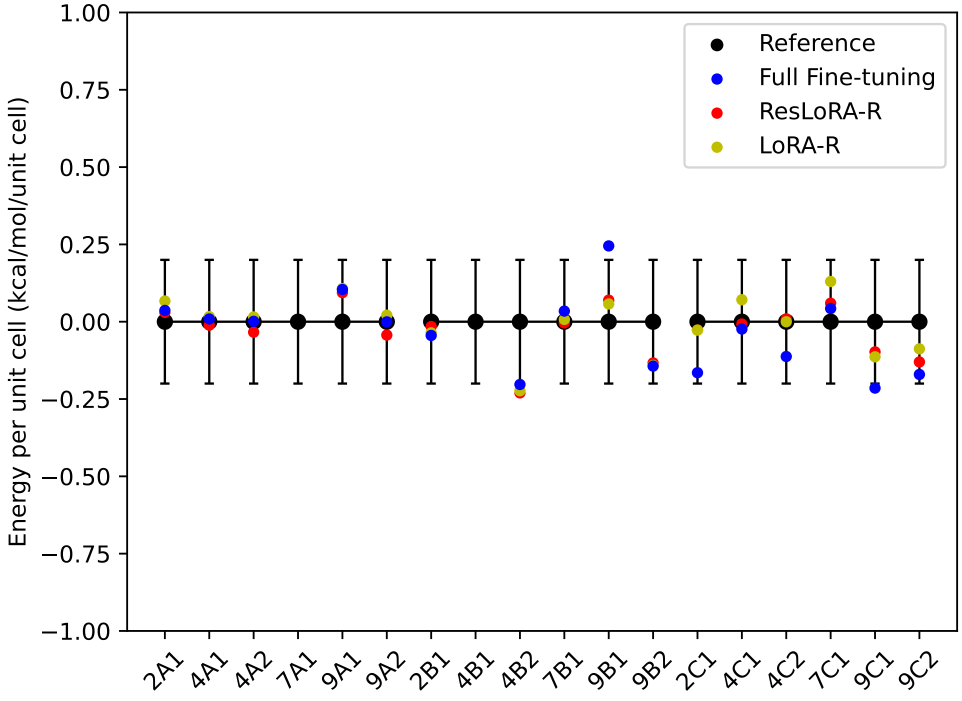

Figure S8: Prediction errors for ice-XV supercells (kcal/mol/unit cell) with full-FT, LoRA-R and ResLoRA-R for various hydrogen orderings.

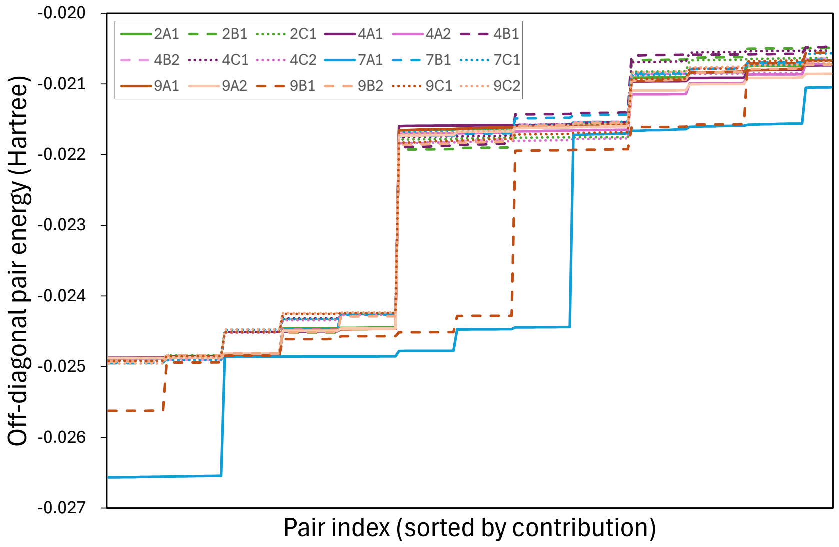

Figure S9: Reference pair energy plots of different ice-XV supercell structures. The most significant 200 off-diagonal pairs for each structure are shown. It is apparent that ice-XV 7A1 supercell structure has drastically different pair energy distributions and this explains why it is an outlier for prediction.

Architectural analysis

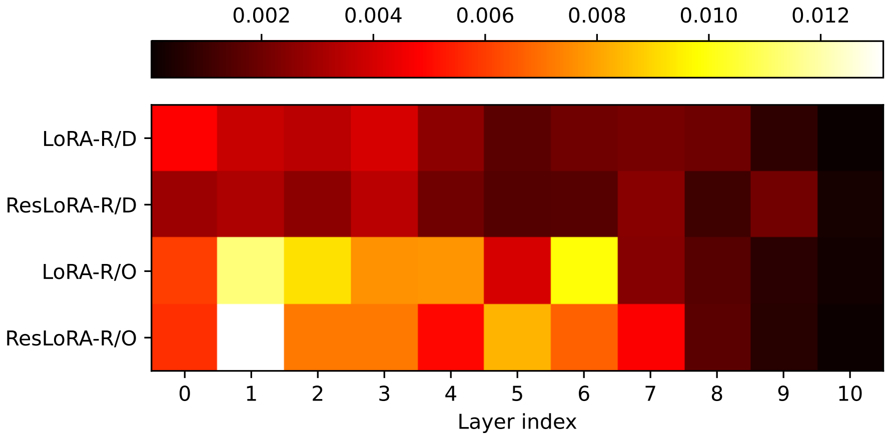

Figure S10: Heat map showing the Frobenius norm ratio $\|\mathbf{\Delta W}\|/\|\mathbf{W_0}\|$ of various hidden layers arranged in sequential order for fine-tuned models on protonated clusters $(H_2O)_{20}(H_3O)^+$ at cc-pVTZ basis set. The layer index 10 corresponds to the linear layer that explicitly learn $\Delta \mathbf{e}_{\mathbf{ij}}^{\mathbf{R}}$. D and O denote diagonal pairs and off-diagonal pairs, respectively.

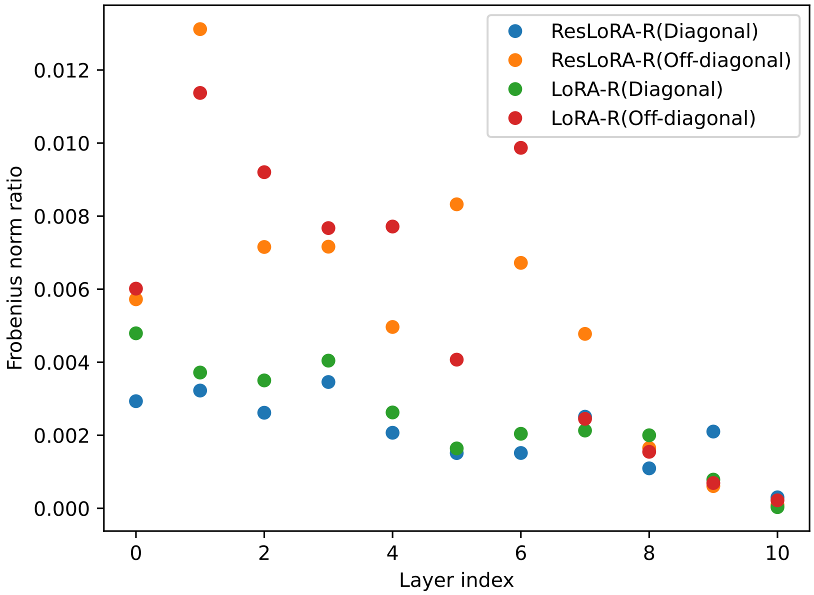

Figure S11: Scatter plot showing the Frobenius norm ratio $\|\mathbf{\Delta W}\|/\|\mathbf{W_0}\|$ of various hidden layers arranged in sequential order for fine-tuned models on protonated clusters $(H_2O)_{20}(H_3O)^+$ at cc-pVTZ basis set. The layer index 10 corresponds to the linear layer that explicitly learn $\Delta \mathbf{e}^{\mathbf{R}}_{\mathbf{ij}}$.

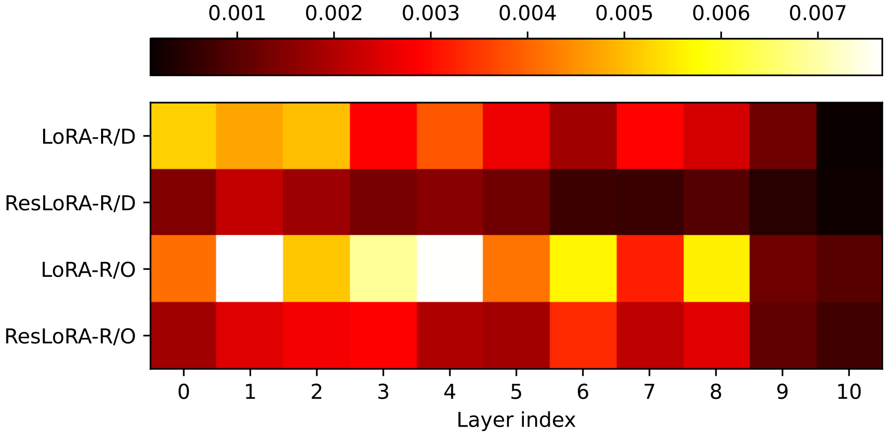

Figure S12: Heat map showing the Frobenius norm ratio $\|\mathbf{\Delta W}\|/\|\mathbf{W_0}\|$ of various hidden layers arranged in sequential order for fine-tuned models on protonated clusters $(H_2O)_{20}(H_3O)^+$ at cc-pVQZ basis set. The layer index 10 corresponds to the linear layer that explicitly learn $\Delta \mathbf{e}^{\mathbf{R}}_{\mathbf{ij}}$. D and O denote diagonal pairs and off-diagonal pairs, respectively.

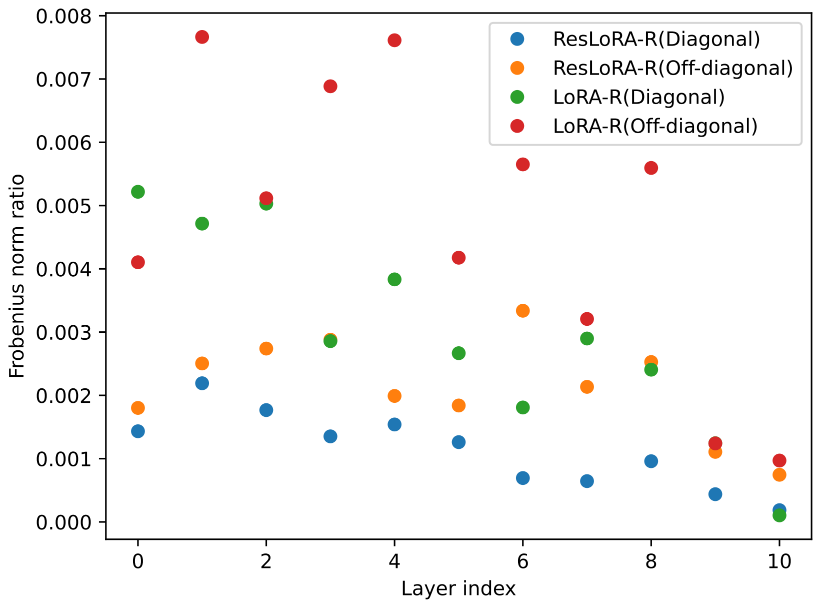

Figure S13: Scatter plot showing the Frobenius norm ratio $\|\mathbf{\Delta W}\|/\|\mathbf{W_0}\|$ of various hidden layers arranged in sequential order for fine-tuned models on protonated clusters $(H_2O)_{20}(H_3O)^+$ at cc-pVQZ basis set. The layer index 10 corresponds to the linear layer that explicitly learn $\Delta e^{\mathbf{R}}_{\mathbf{ij}}$.

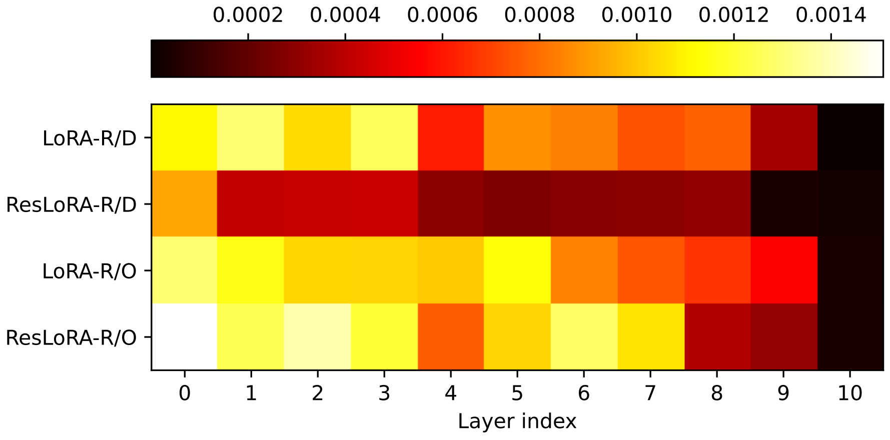

Figure S14: Heat map showing the Frobenius norm ratio $\|\mathbf{\Delta W}\|/\|\mathbf{W_0}\|$ of various hidden layers arranged in sequential order for fine-tuned models on protonated clusters $(H_2O)_{20}(H_3O)^+$ at CCSD(T)/def2-TZVP level of theory. The layer index 10 corresponds to the linear layer that explicitly learn $\Delta e^{\mathbf{R}}_{\mathbf{ij}}$. D and O denote diagonal pairs and off-diagonal pairs, respectively.

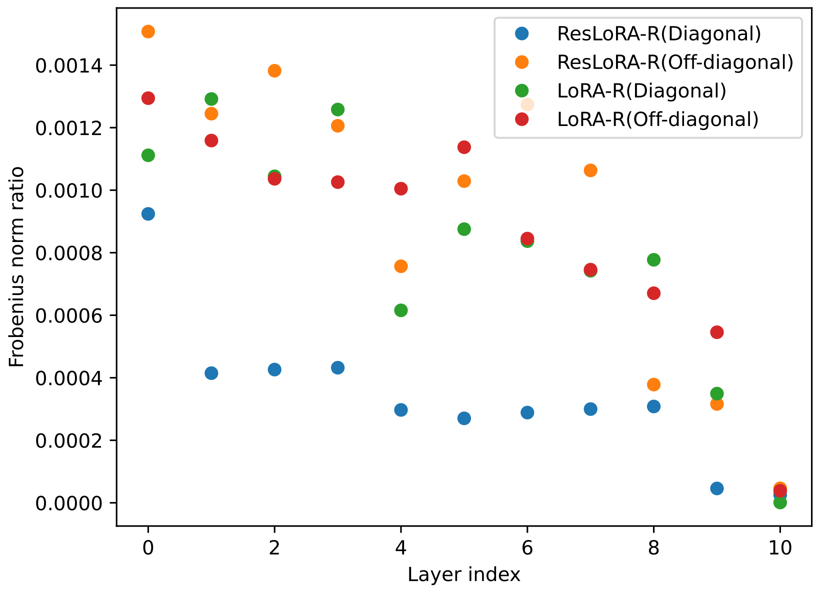

Figure S15: Scatter plot showing the Frobenius norm ratio $\|\mathbf{\Delta W}\|/\|\mathbf{W_0}\|$ of various hidden layers arranged in sequential order for fine-tuned models on protonated clusters $(H_2O)_{20}(H_3O)^+$ at CCSD(T)/def2-TZVP level of theory. The layer index 10 corresponds to the linear layer that explicitly learn $\Delta \mathbf{e}_{\mathbf{ij}}^{\mathbf{R}}$.

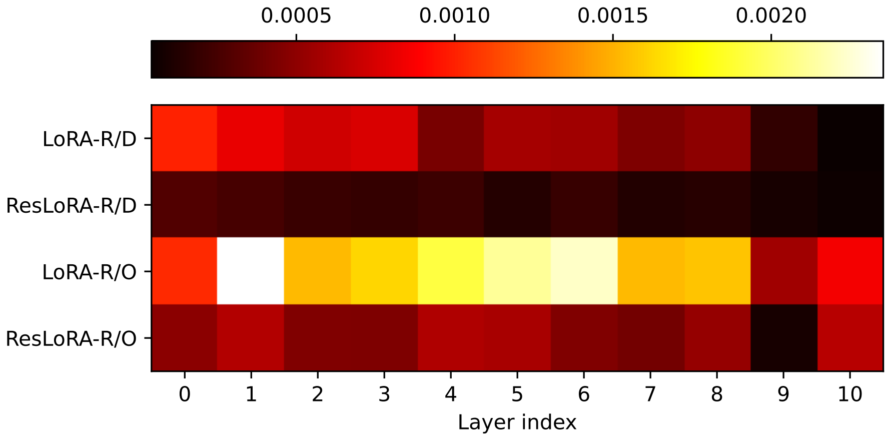

Figure S16: Heat map showing the Frobenius norm ratio $\|\mathbf{\Delta W}\|/\|\mathbf{W_0}\|$ of various hidden layers arranged in sequential order for fine-tuned models on $(Gly)_{10}$ at GTH-TZVP basis set. The layer index 10 corresponds to the linear layer that explicitly learn $\Delta \mathbf{e}_{\mathbf{ij}}^{\mathbf{R}}$. D and O denote diagonal pairs and off-diagonal pairs, respectively.

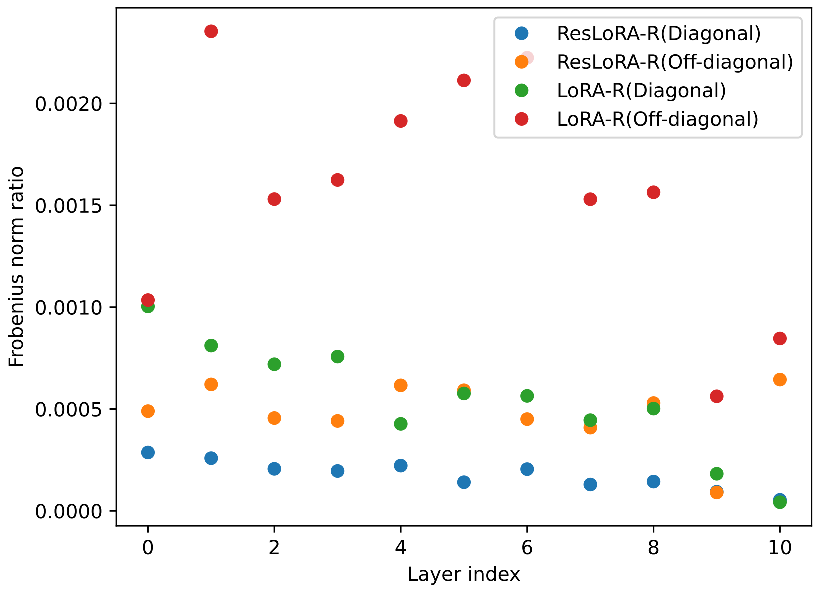

Figure S17: Scatter plot showing the Frobenius norm ratio $\|\mathbf{\Delta W}\|/\|\mathbf{W_0}\|$ of various hidden layers arranged in sequential order for fine-tuned models on $(\mathrm{Gly})_{10}$ at GTH-TZVP basis set. The layer index 10 corresponds to the linear layer that explicitly learn $\Delta \mathbf{e}_{\mathbf{ij}}^{\mathbf{R}}$.

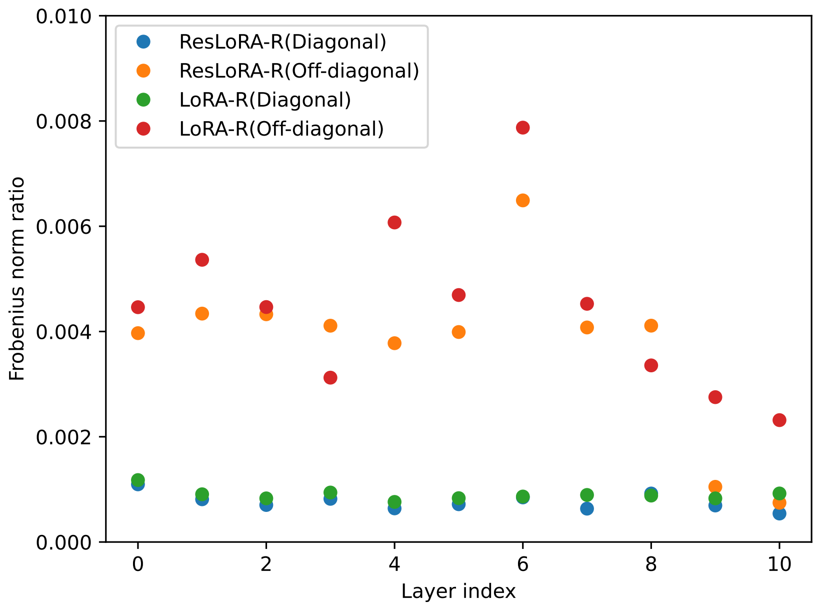

Figure S18: Scatter plot showing the Frobenius norm ratio $\|\mathbf{\Delta W}\|/\|\mathbf{W_0}\|$ of various hidden layers arranged in sequential order for fine-tuned models a single ice XV-4B1 $2{\times}2{\times}2$ supercell structure at GTH-DZVP basis set. The layer index 10 corresponds to the linear layer that explicitly learn $\Delta \mathbf{e}^{\mathbf{R}}_{\mathbf{ij}}$.

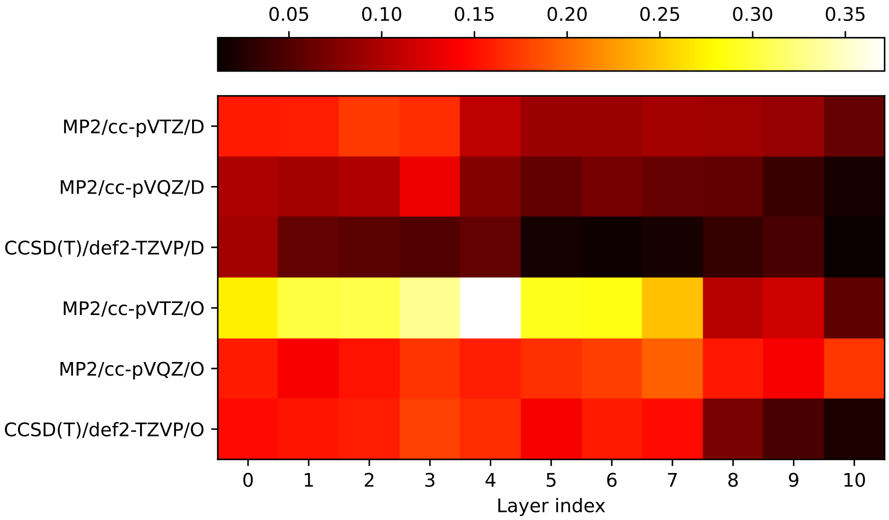

Figure S19: Heat map showing the Frobenius norm ratio $\|\mathbf{\Delta W}\|/\|\mathbf{W_0}\|$ of various hidden layers arranged in sequential order for intermediate models which are fully fine-tuned with 129 small protonated clusters for various theories and basis sets, on a base model pretrained on small $(H_2O)_6$ clusters in periodic boxes with MP2/GTH-DZVP level of theory. D and O denote diagonal pairs and off-diagonal pairs, respectively.

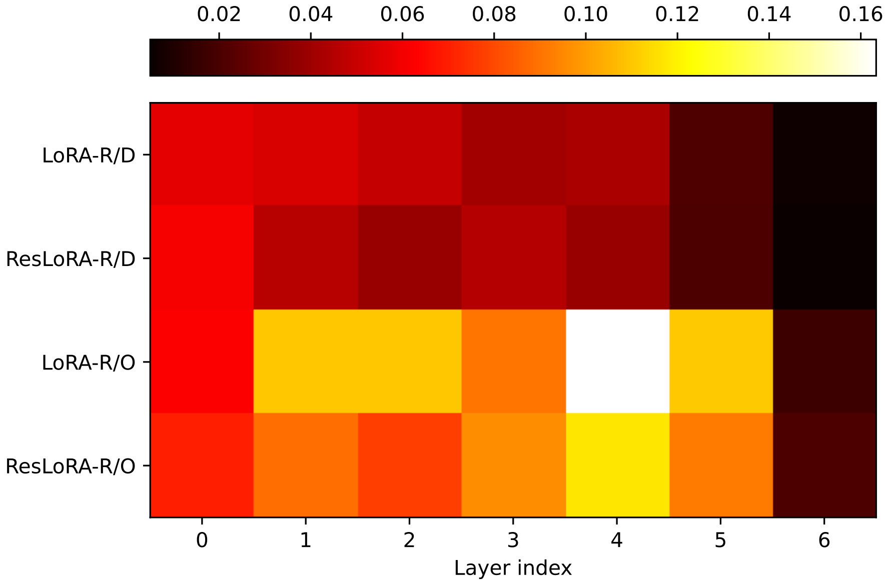

Figure S20: Heat map showing the Frobenius norm ratio $\|\mathbf{\Delta W}\|/\|\mathbf{W_0}\|$ of various hidden layers arranged in sequential order for fine-tuned models on liquid $(H_2O)_{64}$ with 6 hidden layers. The layer index 6 corresponds to the linear layer that explicitly learn $\Delta e_{\mathbf{ij}}^{\mathbf{R}}$. D and O denote diagonal pairs and off-diagonal pairs, respectively.

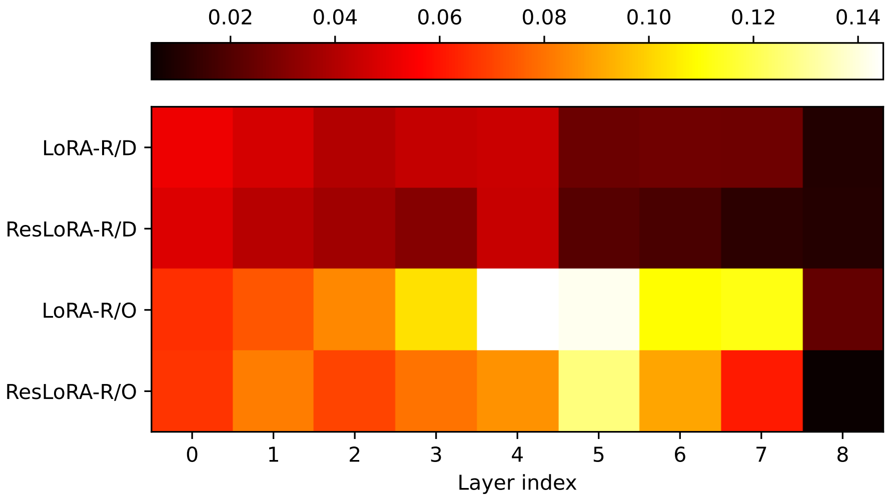

Figure S21: Heat map showing the Frobenius norm ratio $\|\mathbf{\Delta W}\|/\|\mathbf{W_0}\|$ of various hidden layers arranged in sequential order for fine-tuned models on liquid $(H_2O)_{64}$ with 8 hidden layers. The layer index 8 corresponds to the linear layer that explicitly learn $\Delta\mathbf{e}_{\mathbf{ij}}^{\mathbf{R}}$. D and O denote diagonal pairs and off-diagonal pairs, respectively.

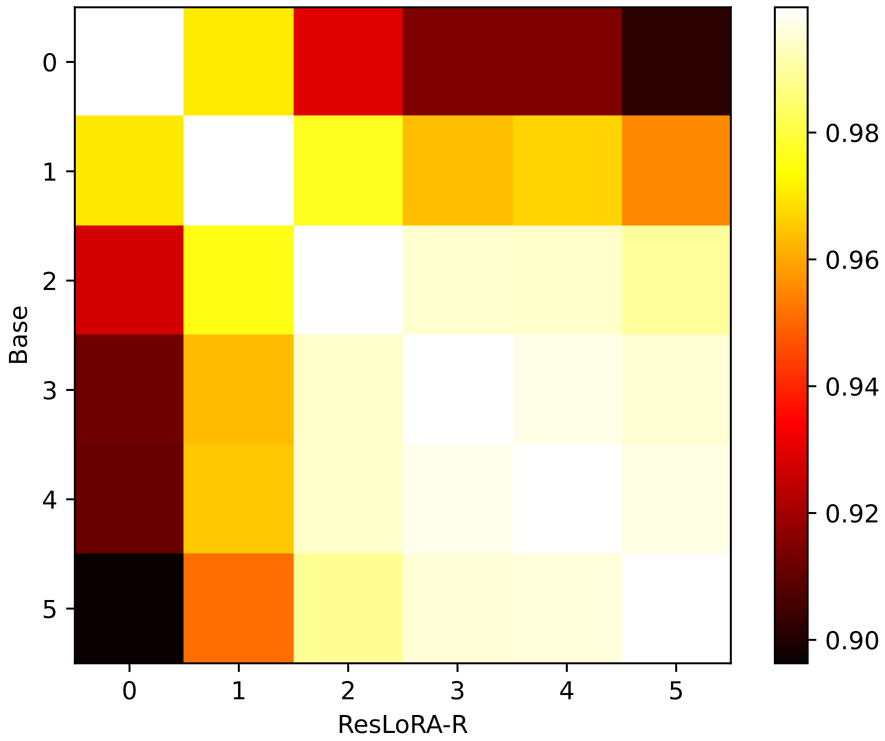

Figure S22: CKA similarity scores for the activations of the diagonal pair base and fine-tuned ResLoRA-R models on liquid $(H_2O)_{64}$ across 6 different hidden layers.

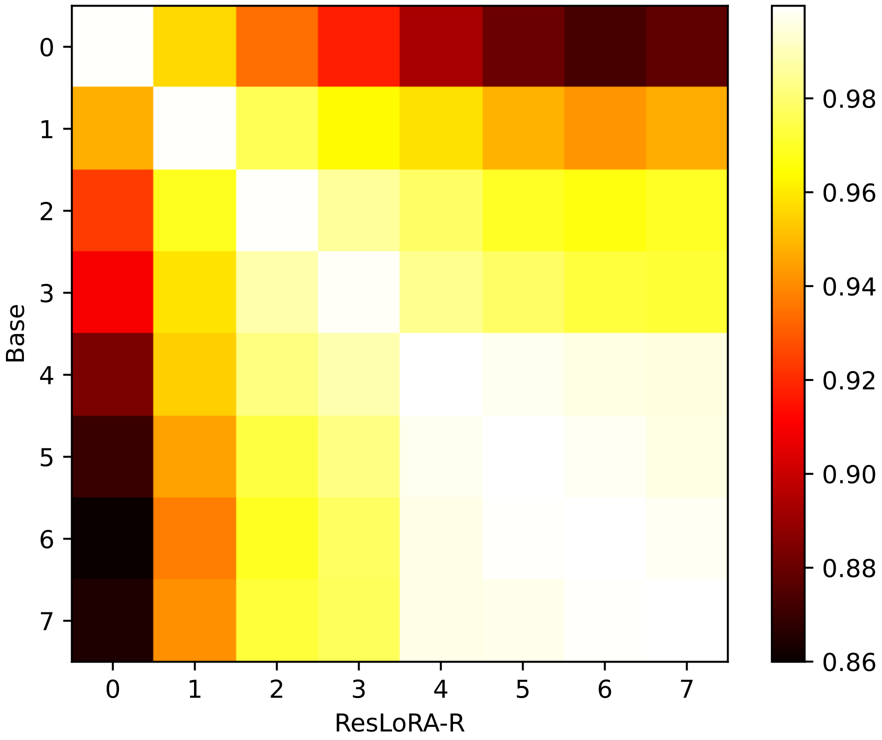

Figure S23: CKA similarity scores for the activations of the diagonal pair base and fine-tuned ResLoRA-R models on liquid $(H_2O)_{64}$ across 8 different hidden layers.

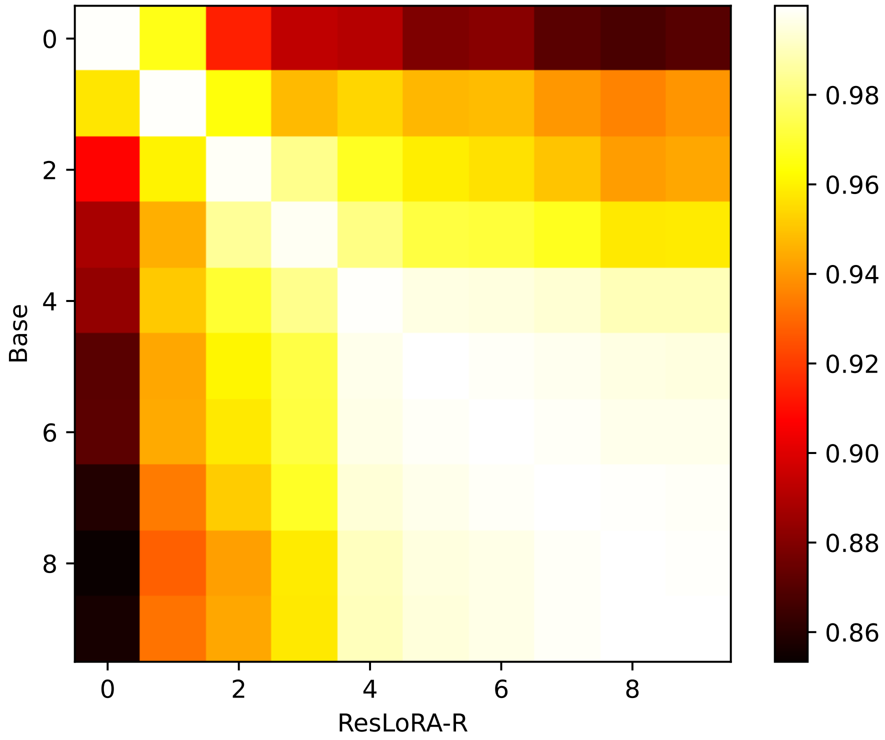

Figure S24: CKA similarity scores for the activations of the diagonal pair base and fine-tuned ResLoRA-R models on liquid $(H_2O)_{64}$ across 10 different hidden layers.

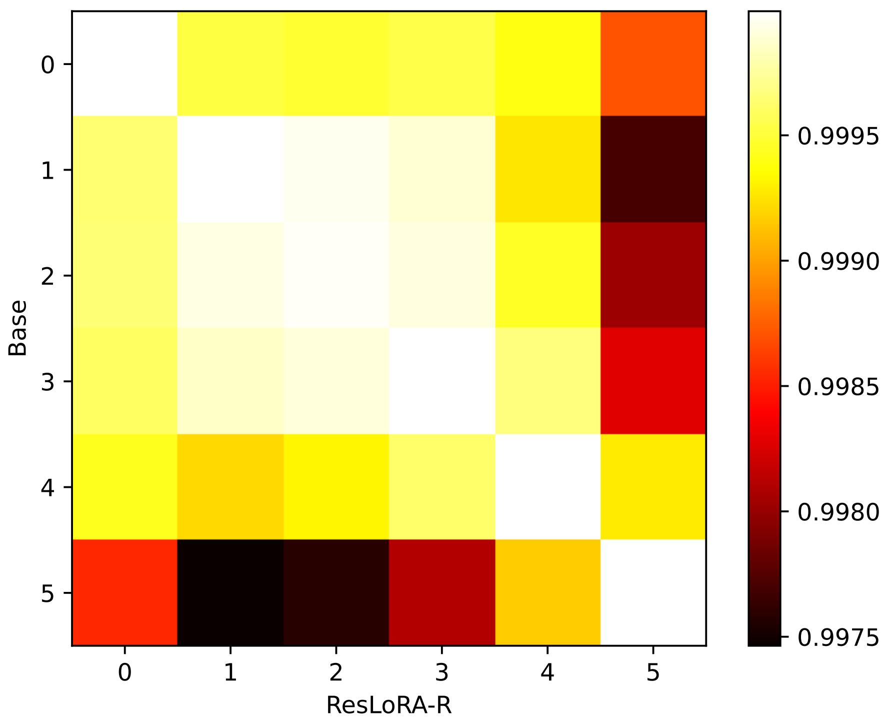

Figure S25: CKA similarity scores for the activations of the off-diagonal pair base and fine-tuned ResLoRA-R models on liquid $(H_2O)_{64}$ across 6 different hidden layers.

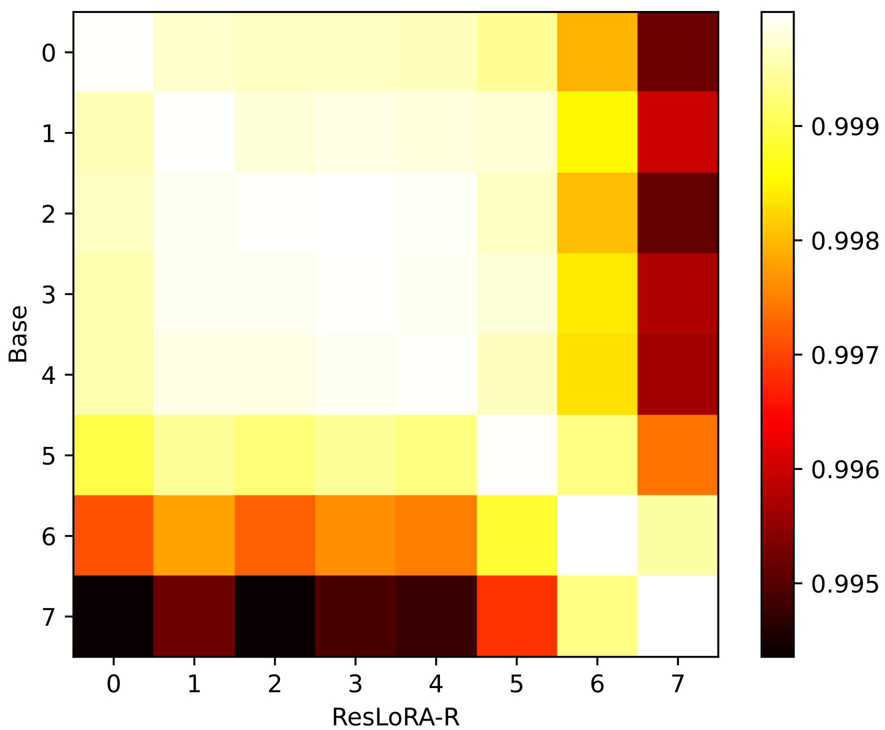

Figure S26: CKA similarity scores for the activations of the off-diagonal pair base and fine-tuned ResLoRA-R models on liquid $(H_2O)_{64}$ across 8 different hidden layers.

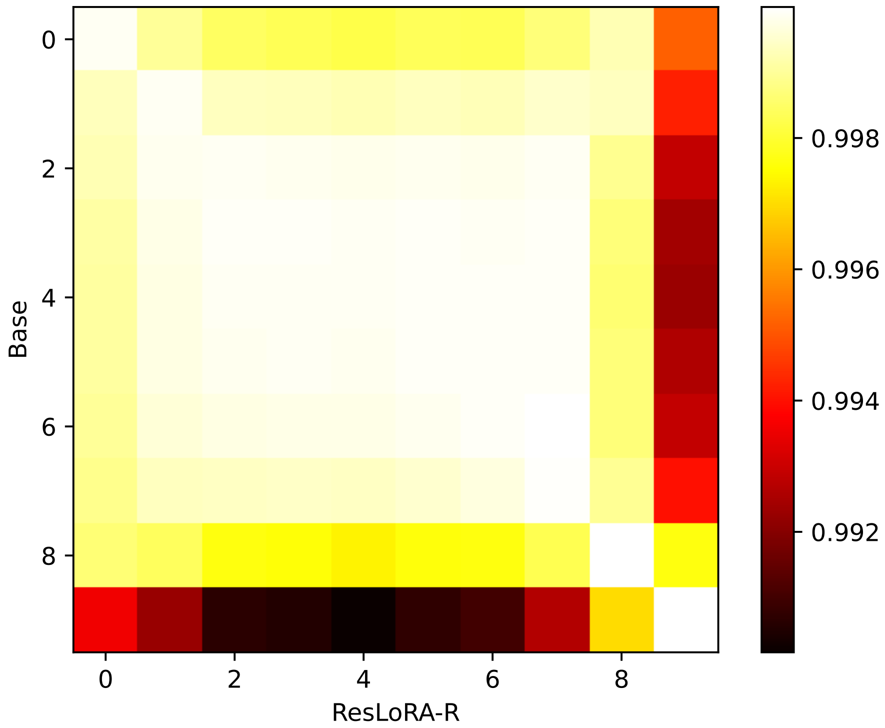

Figure S27: CKA similarity scores for the activations of the off-diagonal pair base and fine-tuned ResLoRA-R models on liquid $(H_2O)_{64}$ across 10 different hidden layers.
